# In-Depth Metaproteomics Analysis of Oral Microbiome for Lung Cancer

**DOI:** 10.34133/2022/9781578

**Published:** 2022-10-13

**Authors:** Xiaoteng Jiang, Yan Zhang, Huiyu Wang, Zeyuan Wang, Shen Hu, Chengxi Cao, Hua Xiao

**Affiliations:** ^1^State Key Laboratory of Microbial Metabolism, Joint International Research Laboratory of Metabolic & Developmental Sciences, School of Life Sciences and Biotechnology, Shanghai Jiao Tong University, Shanghai 200240, China; ^2^School of Pharmacy, Shanghai Jiao Tong University, Shanghai 200240, China; ^3^School of Dentistry and Jonsson Comprehensive Cancer Center, University of California-Los Angeles, Los Angeles 90095, USA; ^4^Department of Instrument Science and Engineering, School of Electronic Information and Electrical Engineering, Shanghai Jiao Tong University, Shanghai 200240, China

## Abstract

The human oral microbiome correlates with numerous diseases, including lung cancer. Identifying the functional changes by metaproteomics helps understand the disease-related dysbiosis, yet characterizing low-abundant bacteria is challenging. Here, we developed a free-flow isoelectric focusing electrophoresis-mass spectrometry- (FFIEF-MS-) based metaproteomics strategy to reduce host interferences and enrich low-abundant bacteria for in-depth interpretation of the oral microbiome. With our method, the number of interfering peptides decreased by 52.87%, whereas the bacterial peptides and species increased by 94.97% and 44.90%, respectively, compared to the conventional metaproteomics approach. We identified 3647 bacterial proteins, which is the most comprehensive oral metaproteomics study to date. Lung cancer-associated bacteria were validated among an independent cohort. The imbalanced *Fusobacterium nucleatum* and *Prevotella histicola* and their dysregulated functions in inhibiting immune response and maintaining cell redox homeostasis were revealed. The FFIEF-MS may serve as a valuable strategy to study the mechanisms between human diseases and microbiomes with broader applications.

## 1. Introduction

The human microbiome plays an important role in maintaining our body homeostasis [1–3]. In particular, the oral microbiome contains nearly 800 species with 20 million nonredundant genes, which is the second most diverse microbiota in the human body and is crucial for connecting the outside environment through the digestive and respiratory tracts [4–6]. Oral microbiome dysbiosis can cause systematic diseases, including lung cancer [7–12], which is the major cause of all cancer deaths worldwide [13]. Majority of these microbiome studies have been conducted through sequencing methods, which provided valuable information on the taxonomic composition and the functional potential of the microbiome [14, 15]. However, changes in functional traits of the microbiome in response to stimuli from the host are the key factor to understand the role of the microbiome in our health and diseases [16–19], which might not be reliably revealed by these methods [20]. The functional analysis relies on the detection of proteins, which is the strength of mass spectrometry-based (meta)proteomics [21, 22]. Therefore, it is of great importance to systematically explore not only the taxonomic composition but also the actual functions of the microbiome that have been expressed under various conditions with metaproteomics. The study of the oral microbiome and lung cancer by metaproteomics may provide more insightful information to characterize the functional role of microbiota than sequencing methods [20], which to our knowledge has yet to be explored.

As a rapidly developing field, metaproteomics has been increasingly applied in studying the gut microbiome [20, 23–26]. However, due to the lack of a comprehensive and standardized metaproteomics workflow, only a few studies have been conducted on the oral microbiome [27–29], even though human saliva is an ideal source for sampling the oral microbiome and showed excellent potential as a noninvasive diagnostic fluid [30–33]. The major challenge is that, due to the relatively high abundance of host interference and low abundance of bacteria within the oral microbiome compared to the gut microbiome, a more dedicated sample pretreatment method is required to improve the efficiency of bacteria identification [34, 35], which was ignored by most of the previous studies [28, 29]. Moreover, current metaproteomics methods may not allow an adequate measurement of low-abundant bacteria in complex microbiomes [36]. The low-abundant bacteria have unneglectable roles in the microbiome community. Based on the “keystone-pathogen hypothesis,,” some low-abundant bacteria could remodel the symbiont microbiome into a dysbiosis community and cause host diseases, while these bacteria remain a minor constituent in the microbiome [37]. In cancer development, certain low-abundant bacteria in our oral and gut microbiome often promote carcinogenesis [38, 39]. Many studies have found the presence of important but low-biomass microbes living in the tumor microenvironment, most of which can promote tumor growth and facilitate tumor cell translocation [40–42]. Besides, there are important functional interactions among the low-abundant bacteria in the microbiome [43]. Losing some of them might lead to a low-diversity microbial community, which is often associated with dysbiosis and diseases, or may even serve as a marker for cancer diagnosis and their prognosis [44, 45]. These features of low-abundant bacteria make them an important and tempting research object in cancer development and warrant further studies. Some studies have introduced peptide-level fractionation prior to mass spectrometry (MS) analysis to increase the number of identified proteins [28, 46]. However, an expanded proteome coverage may not necessarily solve the problem of identifying low-abundant bacteria, since the MS could be saturated by high-abundant proteins' peptides from the host and predominant bacteria [47].

Currently, most method development studies on metaproteomics focus on computational analysis [48–51]. To the best of our knowledge, only a few studies have appreciated the value of sample pretreatment and fractionation that could reduce the complexity of microbiome samples for a comprehensive analysis [52–54]. In our previous work, we used a free-flow isoelectric focusing (FFIEF) electrophoresis method to separate complex microbiome samples and enrich low-abundant bacteria for 16S rRNA sequencing analysis [53]. FFIEF is a liquid-phase preparative separation technique that separates and concentrates biological samples into different fractions based on their isoelectric points (pI), while maintaining their biological activities during the separation [55–58]. However, the host interference remains an impediment for metaproteomics analysis, which calls for a new strategy that integrates optimized sample pretreatment, highly efficient FFIEF separation, and improved data bioinformatics for a comprehensive and in-depth analysis of the human oral microbiome.

In this study, we aimed to develop an FFIEF-MS-based metaproteomics methodology to achieve an in-depth analysis of the human oral microbiome at both the taxonomy composition and the functional level and to provide new insights into the relationship between the oral microbiome and lung cancer. The new strategy consists of three modules: (1) a sample pretreatment module that reduces the host interferences, (2) an FFIEF separation module that fractionates human oral microbiome to enrich the low-abundant bacteria for sensitive identification, and (3) a metaproteomics analysis module that integrates different data analysis methods for integrated functional interpretation [23, 48, 59]. The established strategy greatly increased the numbers of identified bacterial peptides and species, enriched the low-abundant bacteria, and provided a more in-depth functional characterization of human oral microbiome. We further applied this strategy to studying lung cancer-associated dysbiosis at both taxonomic and functional levels. Significantly altered oral bacteria in lung cancer patients were identified and validated, and their dysregulated functions were determined. Our integrated metaproteomic analysis revealed the “key pathogens” that were dysregulated in both abundance and their executed functions in lung cancer.

## 2. Results

### 2.1. Experimental Design

**Figure 1:**
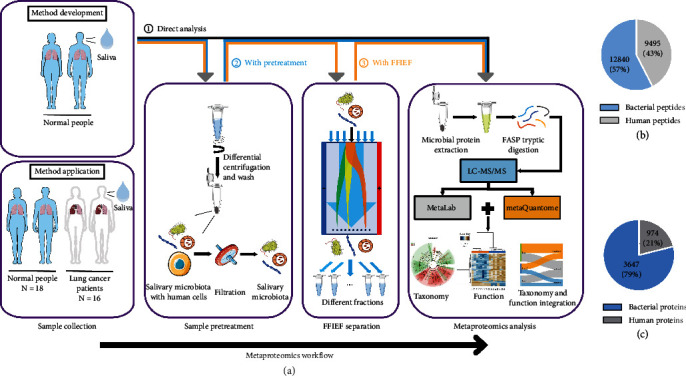
The metaproteomics workflow for the analysis of human oral microbiome. (a) At the method development phase, oral microbiome was processed through: (i) direct Analysis, in which the sample was directly centrifuged to obtain the pellet and extract the proteins for metaproteomics analysis; (ii) with pretreatment, in which the sample was pretreated with differential centrifugation and filtration to remove the interferents from the oral cavity; and (iii) with FFIEF, in which the sample was fractionated by FFIEF to reduce the complexity of microbiome and enrich the low-abundant bacteria for in-depth analysis of the oral microbiome. At the method application phase, the sample went through the optimized FFIEF-MS-based metaproteomics workflow. (b) Total numbers of identified bacterial and human peptides. (c) Total numbers of identified bacterial and human proteins.

In this study, we first developed a metaproteomics strategy, in which the oral microbiome samples were subjected to 3 different workflows: (i) direct analysis workflow (representing the conventional workflow for oral metaproteomics), (ii) pretreatment workflow (similar to the conventional workflow for gut metaproteomics, while we modified it to fit the oral metaproteomics), and (iii) FFIEF workflow (Figure 1(a)). To determine the efficiency of the pretreatment method, samples from 5 healthy subjects were pooled together and then divided into two aliquots for workflows (i) and (ii), respectively. To determine the efficiency of the FFIEF, samples from another 5 healthy subjects were pooled together and then divided into two aliquots for workflows (ii) and (iii), respectively. At the application phase, samples from 18 healthy subjects and 16 lung cancer patients were pooled and went through the established metaproteomics workflow.

In total, we identified 22335 peptides with taxonomy annotation, of which 12840 were bacterial peptides, corresponding to 3647 bacterial proteins. Meanwhile, the numbers of human-originated peptides and proteins were 9495 and 974, respectively (Figures 1(b) and 1(c)).

### 2.2. Microbiome Sample Pretreatment Facilitated the Bacteria Identification via Reducing the Host Interference

**Figure 2:**
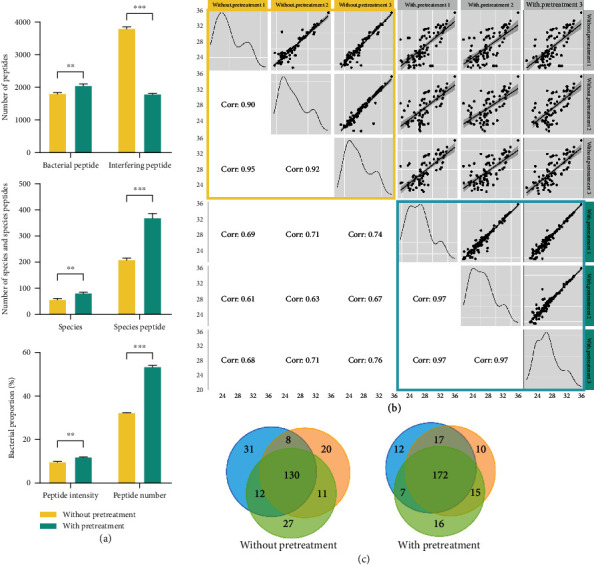
The performance of the sample pretreatment method. (a) The numbers of identified bacterial peptides and other peptides (top), the numbers of identified species and species-level peptides (middle), and the bacterial proportions of peptide intensity and number over the host (bottom). ^∗∗^*p* < 0.01, ^∗∗∗^*p* < 0.001, two-sided unpaired *t*-test. (b) Pearson correlation coefficient of identified bacterial peptides between the methods with or without pretreatment. (c) Venn diagram of the identified bacteria taxa by the methods with or without pretreatment (*n* = 3, three technical replicates, each circle represents a replicate).

Since the oral microbiome sample contained substantial host interferences such as mucin and oral epithelial cells, it is important to remove them so that the low-abundant bacteria could be revealed. By using the direct analysis workflow (i), we identified 1803 bacterial peptides, 3798 interfering peptides, 209 species peptides, and 56 species in an average of three technical replicates. The proportions of bacterial peptide intensity and number over the total peptide were 9.43% and 32.19%, respectively (Figure 2(a)). By using pretreatment workflow (ii), we only identified 1790 interfering peptides, which significantly decreased by 52.87% compared to workflow (i). With fewer identified interfering peptides, the numbers of bacterial peptides, species peptides, and species were increased to 2049, 370, and 81, respectively. The proportions of bacterial peptide intensity and number were also increased to 11.58% and 53.38%, respectively (Figure 2(a)).

Furthermore, the workflow (ii) exhibited a higher reproducibility in bacteria identification than the workflow (i). Pearson correlation coefficients of identified bacterial peptides were 0.97 for pretreated samples versus 0.92 for directly analyzed samples (Figure 2(b)). More bacterial taxa were identified in the pretreated samples than in directly analyzed samples (249 versus 239), and the proportion of taxa overlaps in three replicates was higher in the pretreated samples (69% versus 54%, Figure 2(c)). The relative standard deviations (RSD) of the bacterial taxa that identified in only one technical replicate were 3.06% and 5.57% in workflow (ii) and workflow (i), respectively. Our results demonstrated that the pretreatment method facilitated the identification of oral microbiome with improved reproducibility.

### 2.3. FFIEF-MS Method Allowed for More In-Depth Analysis and Identification of Bacterial Peptides

**Figure 3:**
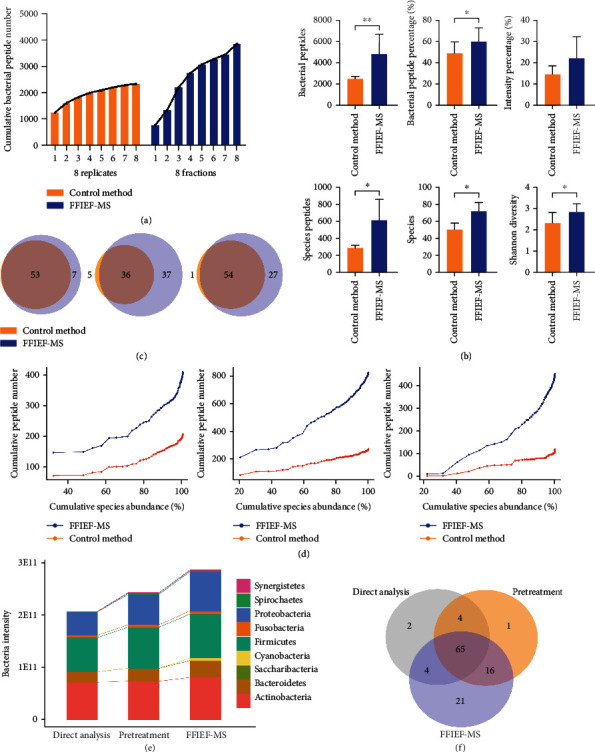
The performance of FFIEF-MS metaproteomics. (a) The number of identified bacterial peptides from the sample without FFIEF separation (8 replicates) and from 8 FFIEF fractions. (b) The identified bacterial peptide number, the proportions of bacterial peptide number and intensity to total peptides (top 3 bar plots), the identified species peptide number, species number, and Shannon diversity (bottom 3 bar plots) from the microbiome samples with the FFIEF-MS method or control method without FFIEF. ^∗^*p* < 0.05, ^∗∗^*p* < 0.01, ^∗∗∗^*p* < 0.001, two-sided paired *t*-test (*n* = 3, three biological replicates). (c) Venn diagrams of the identified bacterial species from oral microbiome samples with the FFIEF-MS method or control method (*n* = 3, three biological replicates). (d) Cumulative number of identified species peptides with the FFIEF-MS method or control method under the cumulative abundance of species arranged from high to low (*n* = 3, three biological replicates). (e) The intensities of bacteria phylum that were identified by direct analysis (workflow (i)), pretreatment only (workflow (ii)), and FFIEF-MS (workflow (iii)). The intensities were calculated by label-free quantification (MaxLFQ algorithm) according to their corresponding peptide intensities. (f) Venn diagram of the identified bacterial species through direct analysis (workflow (i)), pretreatment only (workflow (ii)), and FFIEF-MS (workflow (iii)).

Based on our previously established method [53], we further optimized the experimental parameters and adjusted the instrument for in-depth metaproteomic analysis. Then, the microbial sample was separated through FFIEF after pretreatment (FFIEF-MS, workflow (iii)) to obtain eight fractions (Supplemental Figure 1). Since repeating LC-MS/MS measurements of the same sample could also increase the number of identified peptides, we ran the corresponding sample with LC-MS/MS (without FFIEF, control method, workflow (ii)) eight times for a fair comparison (eight fractions versus eight replicates). In total, 3858 bacterial peptides were identified from the eight FFIEF fractions. However, only 2348 bacterial peptides were identified in the eight replicates, which accounted for 61% of the bacterial peptides identified by the FFIEF-MS approach (Figure 3(a)). Therefore, the increased number of identified bacterial peptides by FFIEF-MS did not simply rely on the increment of MS measuring times.

We further performed two more biological replicates to evaluate the FFIEF-MS method (workflow (iii)). After FFIEF-MS, 4808 bacterial peptides were identified, accounting for 59.59% of the total peptides. Compared to 2466 bacterial peptides identified by the control method (workflow (ii), which accounted for 48.71% of total peptides), the number of bacterial peptides was significantly increased by 94.97% through our FFIEF-MS method (Figure 3(b)). The intensity percentage of bacterial peptides was also increased from 13.49% to 20.47%. At the species level, the identified peptide and species numbers were both significantly increased from 285 peptides and 49 species to 608 peptides and 71 species (113.33% and 44.90% increase, respectively). Shannon diversity was also increased significantly from 2.32 to 2.83 after FFIEF-MS (Figure 3(b)). In addition, the posterior error probability (PEP) value and the peptide searching score distribution of these newly identified bacterial peptides by the FFIEF-MS method showed that these peptides had a low PEP value and comparable searching score with the total peptides, suggesting that the identification of these peptides was highly confident (Supplemental Figure 2). We randomly selected 4 MS/MS spectra of these newly identified peptides and found that all four peptides had satisfactory coverage and intensity of b/y ions, which further demonstrated that these new peptides were reliably identified by our FFIEF-MS method (Supplemental Figure 3).

Moreover, 100% (53 out of 53), 88% (36 out of 41), and 98% (54 out of 55) of bacterial species identified by the control method were also retained by the FFIEF-MS method (Figure 3(c)), which showed only minimum loss in taxonomy after FFIEF. In addition, based on the cumulative curves in Figure 3(d), we found that as the species abundance accumulates, the numbers of identified peptides increased with both methods, while the increment was more obvious with our FFIEF-MS method than the control method, especially from the low-abundant species, indicating that the FFIEF-MS method has an advantage in identifying low-abundant species.

When the intensities of bacteria phylum were compared among the direct analysis (workflow (i)), pretreatment only (workflow (ii)), and FFIEF-MS (workflow (iii)), we found a gradual increase in phylum intensity as each module (pretreatment and FFIEF) was introduced into our metaproteomics strategy (Figures 1(a) and 3(e)). Not only did some high-abundant phyla increase but also the low-abundant phyla were enriched through FFIEF-MS, such as Cyanobacteria and Synergistetes. The total species identified by the three workflows showed that apart from the increased species number identified by the FFIEF-MS (41% increase when compared with the direct analysis and 23% increase when compared with the pretreatment), more than 92% (*n* = 3) of the species were preserved during the FFIEF-MS workflow (Figure 3(f)). Together, these data demonstrated the ability of our strategy to reduce the interferences, enrich the low-abundant species, and improve the sensitivity of microbiome identification, while preserving most species for metaproteomics analysis.

### 2.4. FFIEF-MS Workflow Facilitated a Comprehensive Understanding of Oral Microbiome

**Figure 4:**
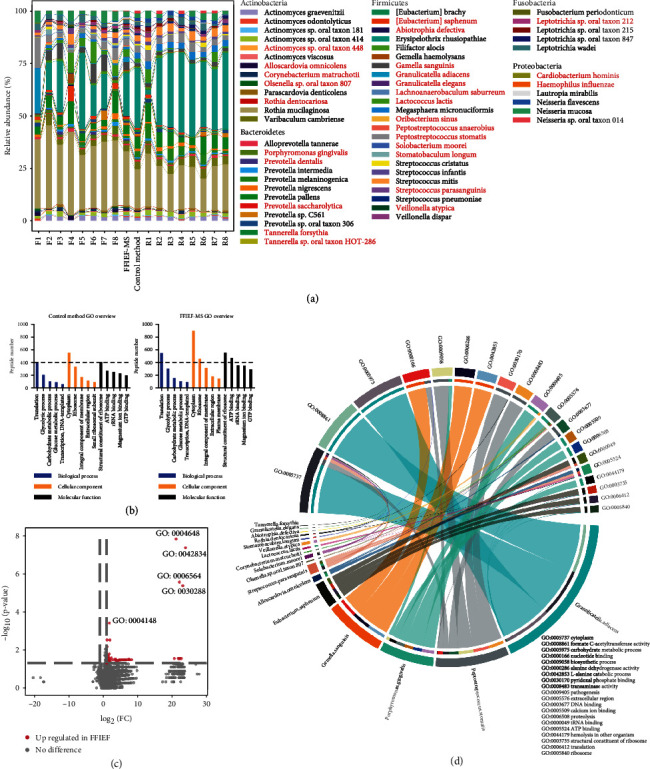
Taxonomic and functional profiling of human oral microbiome samples with the FFIEF-MS method or control method. (a) The species-level composition bar plot of the identified bacteria. F1-F8, 8 FFIEF fractions. FFIEF-MS, combined information from 8 FFIEF fractions. Control method, combined information from 8 replicates (without FFIEF). R1-R8, 8 replicates. Red denoted that species are significantly enriched by FFIEF-MS. (b) Gene Ontology (GO) analysis from the control method and FFIEF-MS. The enrichment level was determined by the number of identified peptides represented in these GO terms. (c) Differential analysis of bacterial functional abundance from FFIEF-MS versus the control method. Each dot represents a GO term. Significantly enriched (red) functions by FFIEF-MS are indicated (|log_2_FC| > 1, *p* value < 0.05). No significantly depleted function was observed. (d) Taxonomy-function integration of the significantly enriched bacterial species by the FFIEF-MS method and their corresponding functions (top 20 GO functions in abundance). Each color represents a species or a GO term. The thickness of each curve corresponds to the relative abundance of each function.

Taxonomy classification of the eight FFIEF fractions (F1-F8, workflow (iii)) and the eight replicates (R1-R8, workflow (ii)) showed that 5 phyla (Actinobacteria, Bacteroidetes, Firmicutes, Fusobacteria, and Proteobacteria) and 60 species were identified by FFIEF-MS (Figure 4(a)). Phylum Firmicutes contained most of the identified species in the oral microbiome with the highest abundance. The low-abundant species, such as *Rothia dentocariosa* and *Streptococcus parasanguinis*, were greatly enriched after FFIEF (red denoted in Figure 4(a) and Supplemental Table S2). The composition of the eight replicates showed a similar pattern; in contrast, the eight FFIEF fractions were very different from each other. In the most acidic fraction F1, *Granulicatella adiacens* and *Peptostreptococcus stomatis* were greatly enriched when compared to other fractions. In the most basic fraction F8, *Porphyromonas gingivalis* and *Peptostreptococcus anaerobius* were enriched. From the three biological replicates, the numbers of significantly enriched species by FFIEF-MS were 27, 25, and 34. Among them, 77% of species were low abundant (abundance < 0.1%) and were masked in the control method (Supplemental Table S3).

Gene ontology (GO) was used for functional annotation of the bacterial peptides. The overviews of their core functions were the same before and after FFIEF fractionation, which means that the functional structure of the microbiome was not changed by FFIEF-MS, while the number of GO annotated peptides was increased (Figure 4(b)). We found that 98 bacterial functions were enriched by our FFIEF-MS method, and no significant depletion of functional annotations was observed (Figure 4(c)), which suggested that no functional information was lost during FFIEF, and no bias was introduced to distort the functional analysis. The functional cluster also revealed the same enrichment trend mentioned above, with F3 and F7 enriching most functional annotations that were not enriched by the control method (Supplemental Figure 4).

We further performed the taxonomy-function integration analysis for the significantly enriched low-abundant species by FFIEF-MS and their corresponding functions (Figure 4(d)). In these species, *Granulicatella adiacens* was the most abundant one, which was responsible for the top 3 abundant functions: cytoplasm, formate C-acetyltransferase activity, and carbohydrate metabolic process, along with other 4 functions. The low-abundant *Peptostreptococcus stomatis* and *Porphyromonas gingivalis* executed diversified functions (9 and 6 GO terms, respectively). For instance, *P. stomatis* was the most activate species in the molecular function, involving nucleotide binding, pyridoxal phosphate binding, transaminase activity, and peroxiredoxin activity (Supplemental Figure 5). *P. gingivalis* was involved in many biological processes that seem to be deleterious, including pathogenesis, proteolysis, and hemolysis in other organism (Supplemental Figure 6). These low-abundant bacteria and their functions revealed by our FFIEF-MS method could not be identified by conventional methods (workflows (i) and (ii)), which therefore stressed the value of our strategy.

### 2.5. Identification of Lung Cancer-Associated Bacteria with FFIEF-MS-Based Metaproteomics

**Figure 5:**
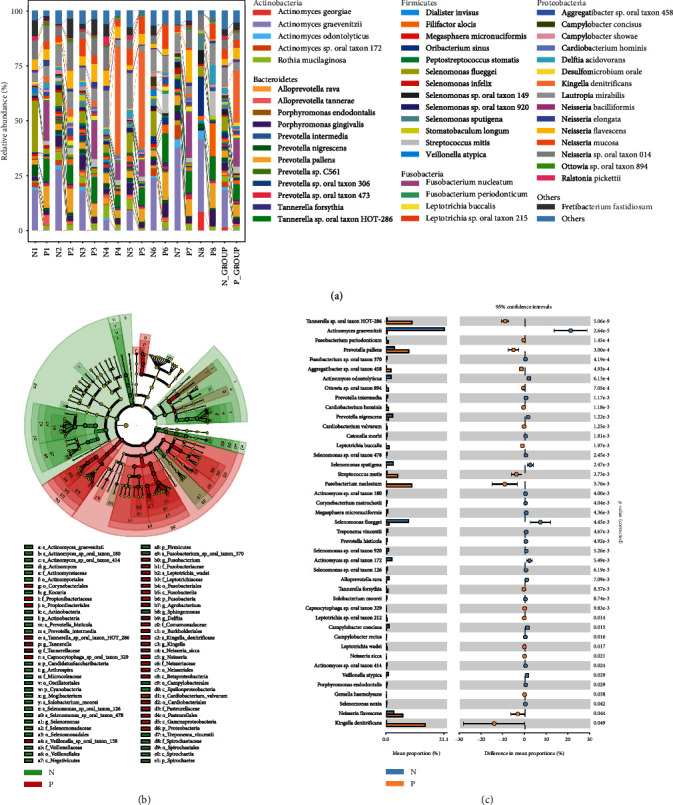
The taxonomic profiling of the oral microbiome samples from lung cancer (P) and healthy (N) groups that were analyzed by the FFIEF-MS-based metaproteomics. (a) The species-level composition bar plot of the identified bacteria (top 50 species) from group P and group N. N1-N8, 8 FFIEF fractions of group N. P1-P8, 8 FFIEF fractions of group P. N_GROUP, combined information from 8 FFIEF fractions of group N. P_GROUP, combined information from 8 FFIEF fractions of group P. (b) LEfSe analysis of the identified bacteria, significantly more abundant in group P (red) and group N (green). The circular cladogram presents the structure and predominant taxa of oral microbiome in each group. (c) Significantly different bacterial species between group N and group P (corrected *p* value < 0.05, Wilcoxon rank sum test, analyzed in STAMP).

Since the oral bacteria can serve as indicators for lung cancer, we applied our FFIEF-MS method to identify lung cancer-associated bacteria through comparing the taxonomy differences between the lung cancer group (group P, *n* = 16, pooled sample) and the healthy group (group N, *n* = 18, pooled sample). Without FFIEF separation, the microbiome diversity in group P was significantly lower than that in group N, which was in accordance with previous studies [60]. While with FFIEF-MS, the microbiome diversity increased significantly in both groups (Supplemental Figure 7). Taxonomic composition revealed drastic differences between group N and group P (Figure 5(a) and Supplemental Table S4). Overall, Actinobacteria and Firmicutes were decreased in group P, while Fusobacteria and Proteobacteria were increased. We then conducted the linear discriminant effect size (LEfSe) analysis to explore marked differences of bacteria between the two groups (Figure 5(b)). Genus *Fusobacterium* and *Neisseria*, family Neisseriaceae and Actinomycetaceae, and order Burkholderiales were characteristic bacteria in lung cancer, while decreased genus *Actinomyces* and class Spirochaetia was found in lung cancer, which corroborates the findings in previous sequencing-based studies [9, 61–66].

**Figure 6:**
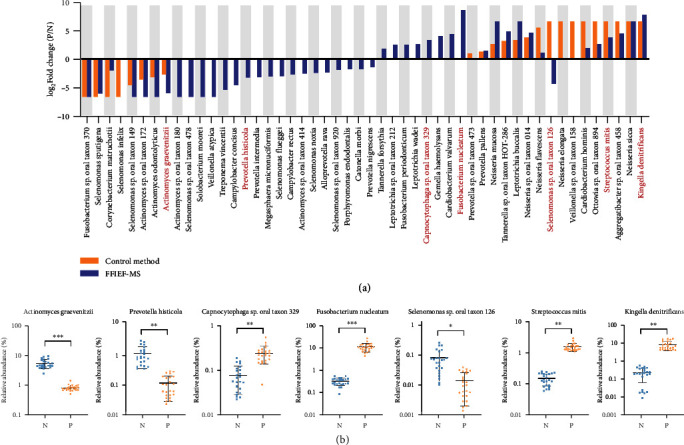
Differences between the lung cancer-related bacteria identified by the FFIEF-MS method and control method and qPCR validation. (a) Fold change of significantly different species (combined results from Figures 5(b) and 5(c)) from group P over group N that were obtained with the FFIEF-MS (with FFIEF) or control method (without FFIEF). Red-colored species were validated by qPCR as shown in Figure 6(b). (b) qPCR validation of the lung cancer-associated bacteria in new cohorts (24 lung cancer patients and 24 healthy subjects). ^∗^*p* < 0.05, ^∗∗^*p* < 0.01, ^∗∗∗^*p* < 0.001, Wilcoxon rank sum test.

Moreover, among the 84 identified species, we discovered 43 significantly different species between lung cancer and healthy groups by our FFIEF-MS method (Figures 5(b) and 5(c)). In comparison, only 24 differential species were identified by the control method without FFIEF.  Figure 6(a) shows the log_2_ transformed fold change (log_2_FC) (P/N) of the significantly different species that were identified with or without FFIEF (43 species in FFIEF-MS, 24 species in the control method). The quantitative differences were more apparent for most species with the FFIEF-MS method. Interestingly, some species identified by the control method showed lower fold changes or even a reversed trend with the FFIEF-MS method. For example, the log_2_FC of *Streptococcus mitis* was reduced from 6.64 to 3.86. *Selenomonas* sp. oral taxon 126 was identified as upregulated in group P by the control method, while it was determined as downregulated by FFIEF-MS (Figure 6(a)). The reason was that *S. mitis* was a low-abundant species in healthy people, which was masked in the group N and solely identified in the group P by the control method. Therefore, the fold change (P/N) was determined as more than 100, corresponding to 6.64 in log_2_FC. Since *S. mitis* was enriched to a detectable level in group N by FFIEF-MS (0.29%), its log_2_FC was reduced to 3.86. Similarly, *Selenomonas* sp. oral taxon 126 was not found in group N by the control method. With the FFIEF-MS method, this low-abundant species was enriched and revealed in group N, which caused the reversed trend.

To validate the bacteria with significantly different levels, we performed qPCR analysis for them in an independent cohort (24 lung cancer patients and 24 healthy subjects), which confirmed the MS-based identification of the lung cancer-associated bacteria, including the downregulated *Actinomyces graevenitzii* and *Prevotella histicola* and the upregulated *Capnocytophaga* sp. oral taxon 329, *Fusobacterium nucleatum*, and *Kingella denitrificans* in the cancer group (Figure 6(b)). In addition, the highly sensitive qPCR enabled the quantification of low-abundant bacteria and confirmed the presence of these species identified by our FFIEF-MS method. For instance, we quantified the *Selenomonas* sp. oral taxon 126 (0.09%) and *S. mitis* (0.17%) in healthy subjects by qPCR, both of which could not be detected by the control method, while both were enriched by our FFIEF-MS method (0.47% and 0.29%, respectively). The qPCR analysis further demonstrated the downregulation of *Selenomonas* sp. oral taxon 126 and the upregulation of *S. mitis* in the cancer group, which validated our findings from the FFIEF-MS (Figures 6(a) and 6(b)). Our results indicated that FFIEF-MS-based metaproteomics facilitated the identification of lung cancer-associated bacteria, and it may eliminate the inappropriate association caused by inadequate measurement of low-abundant species in a complex microbiome with the conventional metaproteomics method.

### 2.6. Functional Analysis and Taxonomy-Function Integration of the Lung Cancer-Associated Oral Microbiome

**Figure 7:**
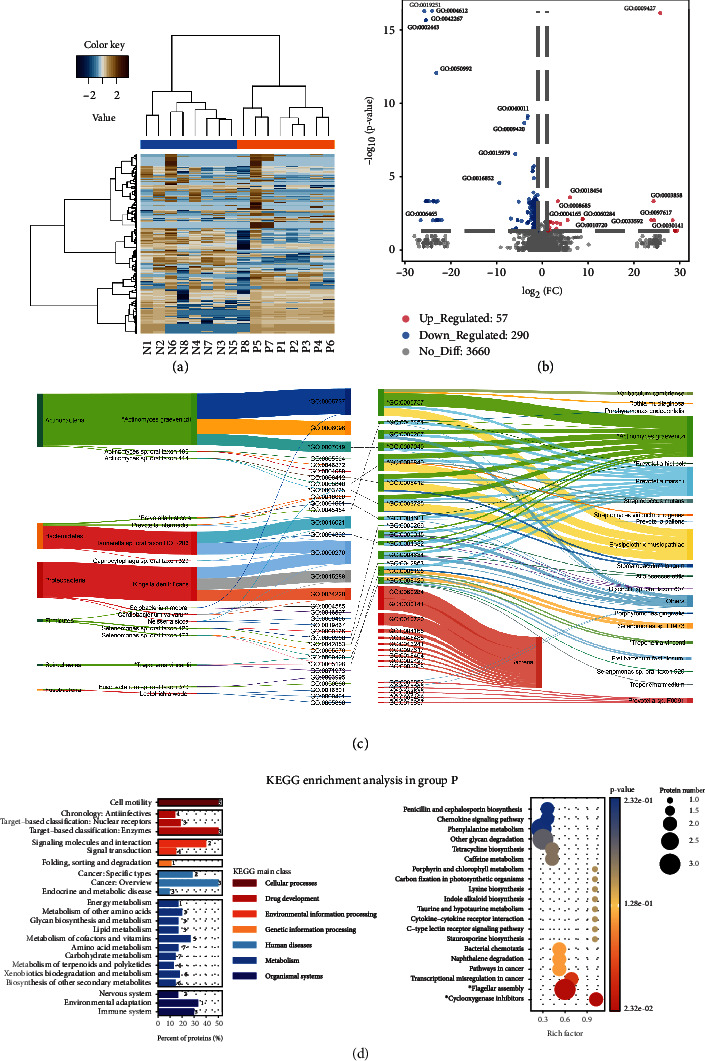
The functional analysis and taxonomy-function integration of lung cancer (P) and healthy control (N) groups. (a) Hierarchically clustered heat map of bacterial function of the lung cancer group (P1-P8, orange) and healthy control group (N1-N8, blue). Each row represents a GO term. (b) Differential analysis of bacterial functional abundance from group P versus group N. Each dot represents a GO term. Significantly upregulated (red) and downregulated (blue) functions in group P are indicated (|log_2_FC| > 1, *p* value < 0.05). (c) Taxonomy-function integration of significantly different species and functions between group P and group N. The left Sankey plot shows the significantly different species (upregulated in red, downregulated in green) and their corresponding functions (top 3 GO terms). The right Sankey plot shows the significantly different functions (top 15 most representative functions in each group, group P in red, group N in green) and their corresponding species. If a GO term could not be specified into a species, it was classified as a bacterial function. The GO term marked by “^∗^” means that this GO function is carried out by not only the significantly different species but also a significantly different function by itself. The thickness of each curve corresponds to its relative abundance. (d) KEGG enrichment analysis in the lung cancer group (group P). The left bar plot shows the enriched pathways at the secondary level. Numbers on the graph represent the identified proteins belonging to the respective group. The percent of proteins means the proportion of identified proteins compared with inputted background proteins. The right bubble plot shows the enriched pathways at the tertiary level. The Rich factor represents the enrichment level.

In the function analysis, 57 GOs were upregulated and 290 GOs were downregulated in the lung cancer group (Figures 7(a) and 7(b)), in which the bacterial cell development and mobility increased in lung cancer, such as the bacterial-type flagellum basal body, distal rod, L ring (GO:0009427), and regulation of cell development (GO:0060284). Meanwhile, the immune-related functions were decreased, such as leukocyte-mediated immunity (GO:0002443) and natural killer cell-mediated cytotoxicity (GO:0042267).

To gain more insights into the relationship between the imbalanced bacteria and the dysregulated functions, we integrated the taxonomic and functional annotations from our metaproteomics data.  Figure 7(c) shows the significantly different species between lung cancer and healthy control groups with their top 3 abundant functions (left) and the significantly dysregulated functions with their corresponding executors (right). We found that *A. graevenitzii* was responsible for cytoplasm (GO:0005737), glycolytic process (GO:0006096), and cell cycle (GO:0007049), in which the cytoplasm and cell cycle (marked by “^∗^”) were also the significantly downregulated functions executed by the imbalanced species like *Solobacterium moorei*, as well as some unchanged species such as *Prevotella marshii* (Figure 7(c), right). Besides, the low-abundant *P. histicola* was involved in two dysregulated functions (peroxidase activity, GO:0004601, and cell redox homeostasis, GO:0045454) and it showed consistency in the taxonomy-function integration, suggesting that it was also the main executor of these two functions. The diminished level of its abundance and corresponding functions could be an important indicator that reflects the imbalanced redox environment in lung cancer [67].

However, most of the species that were predominant in the lung cancer group executed basic functions without significant difference, such as the cell outer membrane (GO:0009279), porin activity (GO:0015288), and ion transmembrane transport (GO:0034220) performed by *K. denitrificans* and *Cardiobacterium valvarum* (Figure 7(c)). In addition, most of the top 15 significantly elevated functions in lung cancer belong to the higher taxonomy level, including regulation of cell development (GO:0060284) and regulation of macroautophagy (GO:0016241) (Figure 7(c)), which implies that these dysregulated functions were executed by multiple homogeneous species with synergistic effect on the disease that could not be specified at the species level [8].

The KEGG enrichment analysis showed that cell motility and cancer-related categories were enriched in the lung cancer group, and the significantly enriched pathways were cyclooxygenase inhibitors and flagellar assembly (Figure 7(d)). In the healthy control group, the microbial metabolism was more diverse than the lung cancer group (Supplemental Figure 10). Besides, the environmental adaptation pathway was solely enriched in the lung cancer group, indicating that there might be disrupted homeostasis between the microbiome and the host in lung cancer. We further constructed the metabolic pathways by mapping our identified bacterial proteins to KEGG, which showed that the healthy control group had much higher coverage of the metabolic pathways than the lung cancer group (Supplemental Figure 8). The fatty acid metabolism pathway was selected for further demonstration, since studies have shown that the metabolic activities of the oral microbiome may be involved in carcinogenesis by regulating obesity and obesity-induced inflammation [3]. The utilization of malonyl-CoA for fatty acid synthesis was significantly decreased in lung cancer, including the complete steps of fatty acid biosynthesis initiation and elongation from malonyl-CoA to stearoyl-CoA in cytoplasm (Supplemental Figure 9). The fatty acid synthesis with the acetyl-CoA module and beta oxidation with the hexadecanoyl-CoA degradation module were enriched in the lung cancer group. Our results suggested a defected fatty acid biosynthesis pathway involving malonyl-CoA and a potentially accelerated fatty acid oxidation process in the microbiome of lung cancer patients.

## 3. Discussion

Given the importance of human oral microbiome in maintaining health and indicating diseases, a thorough study aimed at exploring its taxonomy/function correlation by metaproteomics is needed. When compared with sequencing-based methods like metagenomics and metatranscriptomics, metaproteomics provides valuable mechanistic insights through deciphering the executors of biological functions—proteins—from the host and microbiome [68, 69]. However, the oral microbiome is interfered by the substantial amount of host proteins that may saturate MS analysis, which requires a comprehensive strategy to improve its identification efficiency.

In this study, we developed an FFIEF-MS based metaproteomics methodology to achieve a more in-depth analysis of the oral microbiome. Our strategy can reduce the host interference, enrich the low-abundant bacteria, and integrate the data from both taxonomy and function levels. In total, we identified 12840 bacterial peptides corresponding to 3647 bacterial proteins. To the best of our knowledge, this is the most comprehensive metaproteomics study on the oral microbiome. In previous studies, the numbers of identified bacterial proteins were around 1000 to 2600, with direct analysis of saliva or pelleted saliva [27–29]. The predominant host proteins (e.g., mucin, amylase, and proteins from oral epithelial cells) greatly interfered with the identification of bacterial proteins. In our pretreatment module, we effectively removed the host cells and proteins. The number of interfering peptides was drastically decreased by 52.87%, which led to a significantly increased number of identified bacteria and better reproducibility. Therefore, reducing the host interference was necessary to improve the sensitivity and to achieve a more reliable identification of the microbiome.

By incorporating the FFIEF to separate the complex microbiome and enrich the low-abundant species to a detectable level, we further improved the efficiency of bacterial identification and revealed diversified functions of these low-abundant species. Since some specific low-abundant species may have dominant effects on aggravating dysbiosis in disease [37], it is critical to enrich and identify them. In this regard, FFIEF-MS has unique strength in achieving an in-depth analysis of microbiomes. It should be noted that this improvement was not achieved by simply increasing the MS measuring time. We analyzed one microbiome sample by using two methods in parallel, one with the FFIEF-MS method (8 FFIEF fractions) and the other with the control method (without FFIEF, 8 replicates). The identified bacterial peptides were nearly doubled by using the FFIEF-MS method. Besides, the seemly unsaturated trend of these FFIEF fractions makes it possible for identifying more bacterial peptides if we could add more FFIEF fractions to the MS analysis. However, it would also significantly increase the analysis time and cost for a single experiment. It is a tradeoff between the efficiency and cost that should be considered, which led us to use 8 FFIEF fractions in this study. Moreover, the taxonomy and functional annotations are well retained and further enriched by the FFIEF-MS method, which demonstrates that FFIEF does not damage the bacteria during separation and no bias was introduced prior to downstream MS analysis.

Lung cancer is the leading cause of all cancer deaths worldwide, with a low 5-year survival rate and sometimes poor immunotherapy outcome [13, 70]. It is important to detect lung cancer in a convenient and noninvasive manner and to explore the mechanisms of its development. Human saliva is an easily accessible body fluid for disease diagnostics, and lung cancer patients' saliva contains oral microbiota that were distinct from healthy people [7, 9, 30, 71], making it an ideal medium to reveal lung cancer-associated bacteria and their functions. Although increasing evidence has linked microbiome to lung cancer, most studies were focused on the taxonomic imbalance and the functional interpretation of the microbiota is still lacking [72].

With our established metaproteomics platform, we identified several lung cancer-associated bacteria from the genus to the class level that is consistent with previous sequencing-based studies [9, 61–66] and revealed novel lung cancer-related bacteria that were underestimated by conventional methods. Through an independent cohort validation, 7 species were confirmed to be lung cancer associated. We then found the increased cell development level of bacteria and the decreased immune-related functions in lung cancer. Although the long-term immune response and chronic inflammation are associated with carcinogenesis, increasing evidences suggest that the microbiome can shape the adaptive immunity to escape from immune surveillance [67]. Besides, immune defects may lead to microbiome-driven carcinogenesis and bacteria translocation [3, 73]. Our study also revealed a significantly enriched flagellar assembly pathway in lung cancer, which represents an increased level of bacterial migration and supported the abovementioned finding. In addition, we found that cancer-related pathways, cell motility, and the cyclooxygenase (COX) inhibitor pathways were enriched in the lung cancer microbiome. COX is involved in the synthesis of protective human mucosa [74]. Inhibiting its activity may lead to barrier failure, bacterial translocation, and microbiome-driven carcinogenesis [3], which reflected the potential impact of an imbalanced microbiome on lung cancer development.

Notably, to the best of our knowledge, it is the first time that the upregulated species *F. nucleatum* and its association with the downregulated natural killer cell-mediated cytotoxicity were identified in lung cancer patients. *F. nucleatum* has been reported to directly inhibit natural killer cell-mediated cytotoxicity in colorectal cancer [75]. It promotes cancer development via the virulence factor FadA to invade cells and interact with E-cadherin to activate the beta-catenin signaling pathway [76]. The same pathway was also involved in lung cancer metastasis [77], which may reflect the mechanistic similarity of the two immune-related cancer and explain the association. However, the causal relationship between *F. nucleatum* and lung cancer has not been established. Future studies are warranted to further investigate what role it plays in lung cancer development.

By the integrated analytical method, we can determine the function of the whole microbiome as well as specify the functions to their bacterial executors. Linkages between taxonomy and function were determined with the following insights informed: (1) some dysregulated bacteria indeed execute disrupted functions, (2) some other dysregulated bacteria did not execute the disrupted functions that lead to disease, and (3) some disrupted functions were facilitated by the unchanged bacteria that might be underestimated by the sequencing-based “taxonomy-only” methods. As indicated by our integrated analysis, the dysregulated bacteria that only execute the unchanged functions may not serve as the true indicator to differentiate health and disease [20]. On the other hand, some unchanged bacteria that execute the disrupted functions could be insightful, because their roles in the microbiota may change during disease processes. Accordingly, Hajishengallis et al. showed that some low-abundant bacteria can remodel the normally benign microbiota into a dysbiotic one to facilitate diseases, while their abundance may not change [37]. More importantly, the identified bacteria with dysregulated abundances and functions may serve as valuable biomarkers to diagnose diseases as well as to study the mechanism of the diseases, making them the “key pathogen” for lung cancer study. In a disease-related environment, they change in both numbers and main functions, which means that they might potentially play a key role in the disease and are worth further exploring. For example, we found the downregulated *P. histicola* and its executed functions of peroxidase activity and cell redox homeostasis, which were significantly downregulated functions in the lung cancer group. The cell redox homeostasis is a key indicator for microbiota to maintain its symbiotic relationship with the host [78]. Downregulation of this function could cause dysbiosis and inflammation to the host [79]. It is well known that chronic inflammation could lead to cancer development, including lung cancer [80]. Therefore, the downregulation of its abundance and corresponding redox function could be an important indicator that reflects the imbalanced redox environment in lung cancer [67]. Our method revealed the cancer-associated functional changes in these imbalanced bacteria. Therefore, only focus on the taxonomic changes may not comprehensively identify the disease-associated bacteria, whereas the integration of taxonomy and function can be more informative and reliable.

Currently, most of the studies on lung cancer and microbiome are correlational, in which the causality of microbiome to carcinogenesis remains largely unknown [81]. A previous study has linked the presence of lung microbiota to lung adenocarcinoma via activating *γδ* T cells that produced IL-17 to promote tumor [41]. But they missed the opportunity to explain which bacteria or what bacterial functions activated the *γδ* T cells. With our systematic metaproteomic analysis of microbiomes of lung cancer patients, future studies are warranted to focus on the dysregulated functions and their executors, whether the taxonomic compositions are significantly different or not, and to mine the causal relationships between microbiome and lung cancer in large cohorts.

## 4. Conclusions

In summary, we developed an FFIEF-MS-based metaproteomics strategy that significantly reduced the interference from the host, enriched the low-abundant bacteria, separated the complex microbiota into different fractions to simplify the downstream analysis, and integrated the taxonomy/function analysis. The efficiency of bacterial identification and characterization was significantly improved with good reproducibility by our strategy. We further identified lung cancer-associated bacteria from the phylum to the species level and revealed their dysregulated functions. Seven bacterial species were discovered and validated, which were significantly altered in the lung cancer oral microbiome. Integrated analysis of taxonomy and function revealed that oral bacteria in lung cancer patients were engaged in energy metabolism, reproduction, and migration. Meanwhile, the mutualistic relationship between the host and microbiome was broken. Our data collectively demonstrate that the FFIEF-MS method is a robust and promising strategy in improving the sensitivity of metaproteomics analysis, which has the unique strength in studying the functional perturbations of microbiome in cancer. It may have wider applications in studying the mechanisms between human microbiome and other human diseases.

## 5. Methods

### 5.1. Oral Microbiome Sample Collection

According to the approved protocol (IRB#M15017) by the Institutional Review Board (IRB) of Shanghai Jiao Tong University, unstimulated whole saliva (5 mL) was collected from each study subject in a sterile centrifuge tube on ice, which was followed by centrifugation at 10000 g for 10 min at 4°C to collect the pellet. The sample pellet was stored at −80°C for further use. Lung cancer patients were newly diagnosed and treatment naive at the Shanghai Chest Hospital. Healthy control subjects met the following criteria: no history of chronic pulmonary disease, no respiratory conditions, no oral disease or any type of disease that may influence the oral bacteria (such as chronic inflammation and autoimmune disease), without antibiotic administration in at least three months before sample collection, and with good physical status. Written informed consent was obtained from each human subject. The summarized clinicopathological parameters of lung cancer and healthy subjects are listed in Supplemental Table S1.

### 5.2. Sample Pretreatment

The sample pellets were resuspended in precooled PBS buffer and subjected to sample pretreatment, which consisted of differential centrifugation and filtration steps as inspired by the gut microbiome sample processing to remove most of the large particles, such as host cells and cell debris [23]. The resuspended pellets were centrifuged at 500 g for 5 min at 4°C to collect the supernatants. The remaining pellets were resuspended in cold PBS and washed for two more times. The resulting supernatants were then subjected to a high-speed centrifugation at 16000 g for 20 min at 4°C to collect the pellets, which were further washed with cold PBS and centrifuged again to remove the remaining salivary proteins. After the differential centrifugation, the pelleted bacteria were then resuspended by PBS and filtered through a 5 *μ*m filter to further remove host interferences.

### 5.3. FFIEF Fractionation

The oral microbiome samples after pretreatment were fractionated by FFIEF as previously described with a few modifications [53]. Briefly, the freshly prepared FFIEF carrier buffer (1% ampholyte, 0.5% Triton X-100, 1.1 g/L Ficoll, and 250 mM mannitol in 80 mL ddH_2_O) was injected into the instrument with a constant power of 20 W at 4°C for 1 h to pre-establish a stable pH gradient. Meanwhile, the microbiome samples were incubated in 5 mM precooled CaCl_2_ buffer at 4°C for 20 min. After incubation, samples were centrifuged at 16000 g for 20 min at 4°C and resuspended by FFIEF carrier buffer to perform the electrophoresis fractionation, with the instrument setting changing to a constant voltage of 300 V and a 4 mA current limit for 1 h, with a flow rate of 1.5 mL/min per channel.

After the FFIEF fractionation, thirty-two fractions were collected, which were then combined based on the following rules to simplify downstream analysis: four most acidic fractions 1–4 were mixed as F1, four most basic fractions 29–32 were mixed as F8, and other fractions were crosscombined in which fraction 5, 11, 17, and 23 were mixed as F2; fractions 6, 12, 18, and 24 were mixed as F3; fractions 7, 13, 19, and 25 were mixed as F4, and so on, until fractions 10, 16, 22, and 28 were mixed as F7 (Supplemental Figure 1).

### 5.4. Protein Extraction, Tryptic Digestion, and LC-MS/MS Analysis

The microbiome samples were resuspended in a lysis buffer (4% SDS and 8 M urea in 50 mM Tris-HCl, pH 8.0) with protease inhibitor cocktails (Roche Diagnostics GmbH, German) and subjected to ultrasonication on ice for 5 min (10 s on/off). The lysates were centrifuged at 16000 g for 10 min at 4°C to remove the cell debris. The resulting supernatants were precipitated by 5-fold volume of precooled precipitation solvent (50% *v*/*v* acetone, 50% *v*/*v*ethanol, and 0.1% *v*/*v* acetic acid) at −20°C overnight. The precipitated proteins were pelleted by centrifugation at 16000 g for 30 min at 4°C and washed three times by precooled acetone for desalting. Protein pellets were resuspended by dissolving buffer (6 M urea in 50 mM ammonium bicarbonate buffer), and protein concentrations were determined by the Bradford assay (Thermo Fisher Scientific, USA).

For tryptic digestion, 30 *μ*g proteins were first reduced with 10 mM dithiothreitol (DTT) for 1 h at 37°C and alkylated with 20 mM iodoacetamide (IAA) for 40 min at room temperature in the dark. Then, the filter-aided sample preparation protocol (FASP) was applied to digesting proteins [82], with slight modifications. Briefly, the alkylated proteins were transferred into filtration devices (Sartorius, Germany) and centrifuged at 16000 g for 10 min at 10°C. The proteins were then diluted with 8 M urea and centrifuged. This step was repeated three times. Afterwards, 50 mM ammonium bicarbonate buffer was added to the filtration devices and centrifuged at 16000 g for 15 min at 10°C two times. Trypsin solution (1 : 50 enzyme-to-protein ratio) was added and the samples were incubated at 37°C overnight. After digestion, peptides were desalted using the ZipTip C18 (Millipore, Billerica, MA).

An EASY-nLC 1000 LC system coupled with an Orbitrap Q-Exactive Plus mass spectrometer (Thermo Fisher Scientific, USA) was used for LC-MS/MS analysis with a 120 min gradient from 5 to 35% acetonitrile (*v*/*v*) at a flow rate of 300 nL/min. The mass spectrometer was operated in positive ion mode with an electrospray voltage of 2 kV. The full MS scan was set from 350 to 1500 m/z with the resolution of 70000, followed by data-dependent MS/MS scans of the 20 most intense ions with the resolution of 17500 and a dynamic exclusion duration of 30 s.

### 5.5. Taxonomic and Functional Analysis

The peptide/protein identification, quantification, and taxonomic annotation were constructed by the MetaLab software (version 2.0.0) [48] that searched against the Human Oral Microbiome Database (HOMD) (release 2017_2_16, 2401922 entries) [83] and the Human Uniport database (release 2017_11_28, 20244 entries) [84] with the default settings. This software employs an iterative database search strategy for comprehensive database search with a false discovery rate (FDR) threshold of 0.01, both at the peptide level and at the protein level. It utilizes the MaxLFQ algorithm on MaxQuant for accurate quantification [51]. The identification of total protein was performed based on following rules: unique peptide ≥ 1 and FDR < 1% at both peptide and protein levels [23]. The lowest common ancestor (LCA) method was used for taxonomic assignment, and taxa with equal or greater than 3 distinct peptides were retained for further analysis [23, 52, 85]. The functional analysis was performed with the Unipept (http://unipept.ugent.be) [86] for peptide-based functional annotation and the KofamKOALA (https://www.genome.jp/tools/kofamkoala/) [87] for protein-based functional annotation. The identified protein sequences were extracted from the HOMD database to perform KEGG annotation, and significantly upregulated bacterial proteins (unique peptide ≥ 2, |log_2_FC| > 1, *p* value < 0.05) in lung cancer (216 proteins) and healthy groups (430 proteins) were selected for KEGG enrichment analysis. The taxonomy-function integration was conducted on the Galaxy platform (https://usegalaxy.eu/) [88] using the metaQuantome module [59].

### 5.6. Quantitative Real-Time PCR, Statistical Analysis, and Data Visualization

Quantitative real-time PCR (qPCR) was performed to validate the lung cancer-associated bacteria. The specific primers of each target bacteria and universal primer of the total bacteria were chosen from established works or designed by NCBI Primer BLAST (Supplemental Table S5) [89–91]. The details of qPCR procedures were the same as described previously [53].

The biomass of each taxon was determined by summing the intensity of its corresponding peptides. The LFQ intensities of quantified peptide/protein were log_2_ transformed and used for statistical analysis. Statistical significance was assessed by the two-sided unpaired *t*-test for univariate statistical difference between two groups with Gaussian distribution and by the two-sided paired *t*-test for paired comparison; otherwise, the Wilcoxon rank sum test was used. For two-sample comparison, *G* test (w/Yates') + Fisher's exact test was applied [92]. For multiple comparisons, FDR-correlated *p* values were used. Linear discriminant effect size (LEfSe) analysis was used to determine the significant different taxa between the lung cancer and healthy groups (8 fractions for each group) with the linear discriminant analysis (LDA) threshold greater than 3 [93]. For identifying the lung cancer-associated bacteria, we combined the results from both LEfSe analysis and the STAMP software (Wilcoxon rank sum test) for comprehensive analysis [53, 94].

Bar and violin plots were generated with the GraphPad Prism 8. Taxonomic composition bar plots were generated using iMetaLab (http://imetalab.ca/). Heatmaps with clustering information were visualized using the Galaxy platform (https://usegalaxy.eu/). RStudio was used for Pearson correlation coefficient plot, volcano plots, Circos plots, Venn diagrams, and Kyoto Encyclopedia of Genes and Genomes (KEGG) enrichment plot generation. Sankey plot was generated using SankeyMATIC (http://sankeymatic.com/). KEGG pathway maps were customized by uploading KO numbers to the KEGG website (http://www.kegg.jp) [95].

## Data Availability

All raw data from LC-MS/MS have been deposited to the ProteomeXchange Consortium (http://www.proteomexchange.org) via the PRIDE partner repository (dataset identifiers PXD026727).

## Abbreviations

FDR: False discovery rate
FASP: Filter-aided sample preparation protocol
FFIEF: Free-flow isoelectric focusing
GO: Gene ontology
HOMD: Human oral microbiome database
IBD: Inflammatory bowel disease
IRB: Institutional review board
pI: Isoelectric points
KEGG: Kyoto Encyclopedia of Genes and Genomes
LC-MS/MS: Liquid chromatography–tandem mass spectrometry
LDA: Linear discriminant analysis
LEfSe: Linear discriminant effect size
LCA: Lowest common ancestor.

## Supplementary Materials

Supplementary Materials Figure S1: FFIEF separation scheme and the combination strategy for 32 FFIEF fractions. Figure S2: evaluation of the newly identified bacterial peptide by FFIEF (three biological replicates). A. The histogram of PEP (posterior error probability) value from the newly identified bacterial peptides after FFIEF. B. The density plot of the bacterial peptide score from the newly identified peptides (red) and the total peptides (cyan). Figure S3: the randomly selected MS/MS spectra of 4 newly identified bacterial peptides after FFIEF. A. Peptide that belongs to Fusobacterium. B. Peptide that belongs to Treponema. C. Peptide that belongs to Firmicutes. D. Peptide that belongs to Neisseria. Figure S4: hierarchically clustered heat map of bacterial function from the microbiome sample with/without FFIEF (R1-R8, eight replicates without FFIEF; control: F1-F8, eight FFIEF fractions, FFIEF-MS). Each row represents a GO term. Figure S5: taxonomy-function integration of the significantly enriched bacterial species by FFIEF and their corresponding functions (top 20 abundant molecular functions). Figure S6: taxonomy-function integration of the significantly enriched bacterial species by FFIEF and their corresponding functions (top 20 abundant biological processes). Figure S7: the Shannon diversity of microbiome samples in lung cancer (P) and healthy group (N) before/after FFIEF. Figure S8: the metabolic pathway constructed by identified bacterial proteins. Figure S9: the fatty acid metabolism pathway constructed by identified bacterial proteins. Figure S10: KEGG enrichment analysis in the lung cancer group (group N). Table S1: demographic information of enrolled lung cancer patients and healthy controls. Table S2: taxonomy composition (species level) of microbiome samples with or without FFIEF fractionation. Table S3: the significantly enriched species after FFIEF. Table S4: taxonomy composition (species level) of lung cancer (P) and healthy group (N) in different FFIEF fractions. Table S5: primer sequences for qPCR verification.
