# Supplementary material for: In-Depth Metaproteomics Analysis of Oral Microbiome for Lung Cancer
Source: Research (Wash D C). 2022 Oct 13;2022:9781578. doi: 10.34133/2022/9781578 (PMC9590273; doi:10.34133/2022/9781578)
Supplement: Supplementary Materials — Figure S1: FFIEF separation scheme and the combination strategy for 32 FFIEF fractions. Figure S2: evaluation of the newly identified bacterial peptide by FFIEF (three biological replicates). A. The histogram of PEP (posterior error probability) value from the newly identified bacterial peptides after FFIEF. B. The density plot of the bacterial peptide score from the newly identified peptides (red) and the total peptides (cyan). Figure S3: the randomly selected MS/MS spectra of 4 newly identified bacterial peptides after FFIEF. A. Peptide that belongs to Fusobacterium. B. Peptide that belongs to Treponema. C. Peptide that belongs to Firmicutes. D. Peptide that belongs to Neisseria. Figure S4: hierarchically clustered heat map of bacterial function from the microbiome sample with/without FFIEF (R1-R8, eight replicates without FFIEF; control: F1-F8, eight FFIEF fractions, FFIEF-MS). Each row represents a GO term. Figure S5: taxonomy-function integration of the significantly enriched bacterial species by FFIEF and their corresponding functions (top 20 abundant molecular functions). Figure S6: taxonomy-function integration of the significantly enriched bacterial species by FFIEF and their corresponding functions (top 20 abundant biological processes). Figure S7: the Shannon diversity of microbiome samples in lung cancer (P) and healthy group (N) before/after FFIEF. Figure S8: the metabolic pathway constructed by identified bacterial proteins. Figure S9: the fatty acid metabolism pathway constructed by identified bacterial proteins. Figure S10: KEGG enrichment analysis in the lung cancer group (group N). Table S1: demographic information of enrolled lung cancer patients and healthy controls. Table S2: taxonomy composition (species level) of microbiome samples with or without FFIEF fractionation. Table S3: the significantly enriched species after FFIEF. Table S4: taxonomy composition (species level) of lung cancer (P) and healthy group (N) in different FFIEF fractions. Ta [file 9781578.f1.docx]

Supporting information for

**In-Depth Metaproteomics Analysis of Oral Microbiome for Lung Cancer**

Xiaoteng Jiang ^a, †^, Yan Zhang ^b, †^, Huiyu Wang ^a^, Zeyuan Wang ^a^,

Shen Hu ^c,^*^*^*, Chengxi Cao ^d,^*^*^*, and Hua Xiao ^a,^*^*^*

^a^ State Key Laboratory of Microbial Metabolism, Joint International Research Laboratory of Metabolic & Developmental Sciences, School of Life Sciences and Biotechnology, Shanghai Jiao Tong University, Shanghai, 200240, China

^b^ School of Pharmacy, Shanghai Jiao Tong University, Shanghai, 200240, China

^c^ School of Dentistry and Jonsson Comprehensive Cancer Center, University of California-Los Angeles, Los Angeles, 90095, USA

^d^ Department of Instrument Science and Engineering, School of Electronic Information and Electrical Engineering, Shanghai Jiao Tong University, Shanghai, 200240, China

*^*^* Corresponding Authors:

Dr. Hua Xiao, Email address: [huaxiao@sjtu.edu.cn](mailto:huaxiao@sjtu.edu.cn)

Dr. Chengxi Cao, Email address: [cxcao@sjtu.edu.cn](mailto:cxcao@sjtu.edu.cn)

Dr. Shen Hu, Email address: shenhu@ucla.edu

^†^ These co-first authors contributed equally to this work.

**This supporting information contains:**

Figure S1 to S10

Table S1 to S5

**Supplemental figures**


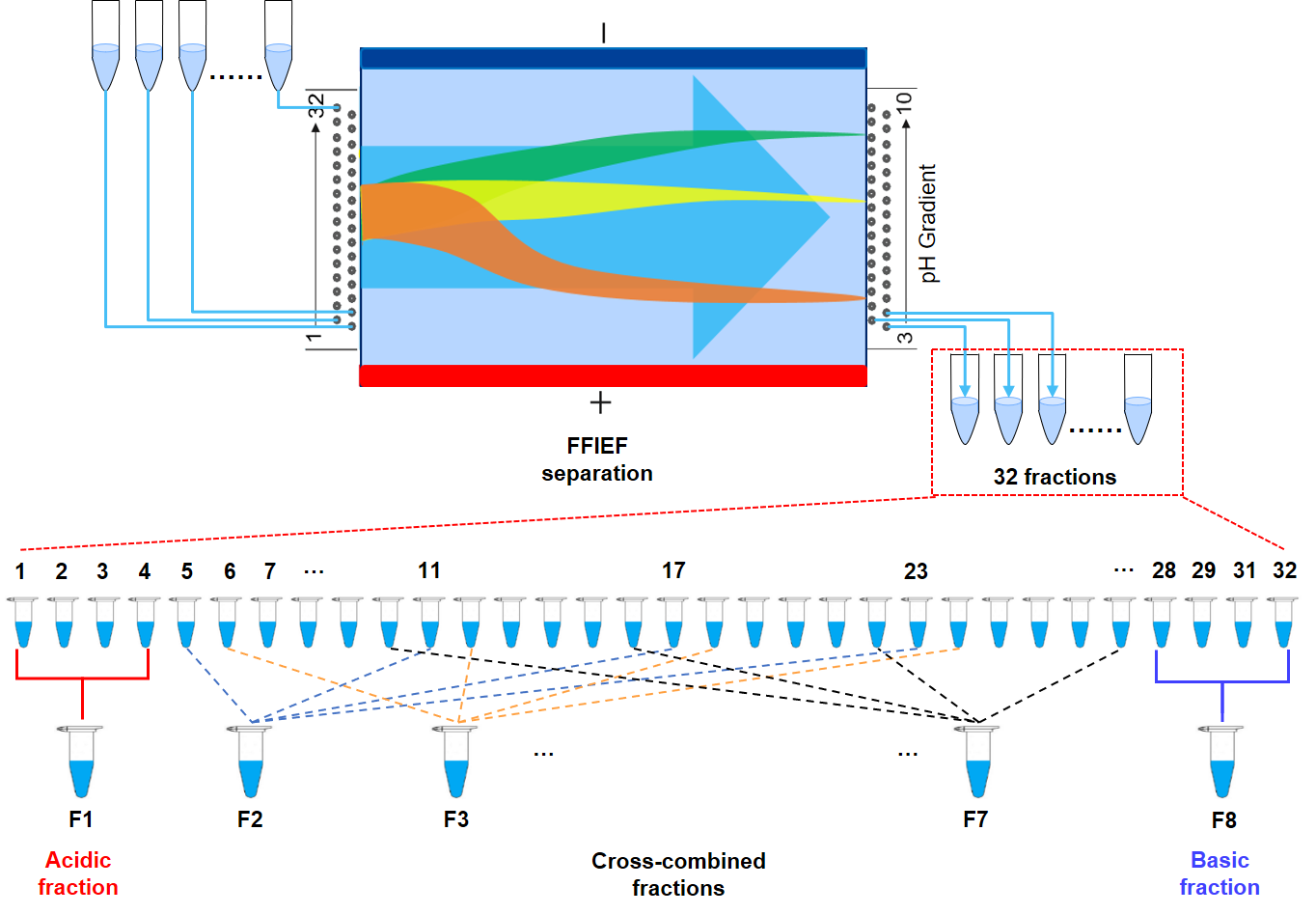


Figure S1. FFIEF separation scheme and the combination strategy for 32 FFIEF fractions.


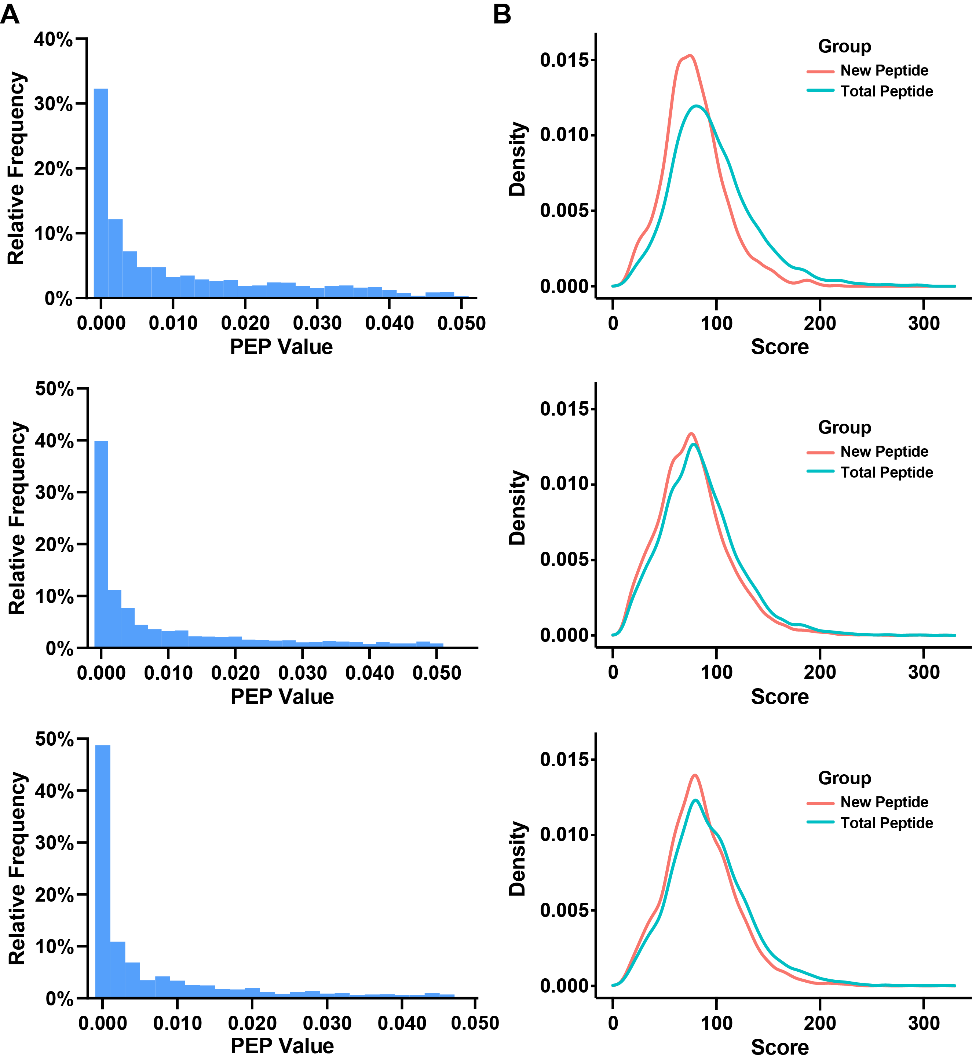


Figure S2. Evaluation of the newly identified bacterial peptide by FFIEF (three biological replicates). A. The histogram of PEP (Posterior Error Probability) value from the newly identified bacterial peptides that after FFIEF. B. The density plot of bacterial peptide score from the newly identified peptides (red) and the total peptides (cyan).

Figure S3. The randomly selected MS/MS spectra of 4 newly identified bacterial peptides that after FFIEF. A. Peptide that belongs to *Fusobacterium*. B. Peptide that belongs to *Treponema*. C. Peptide that belongs to Firmicutes. D. Peptide that belongs to *Neisseria*.


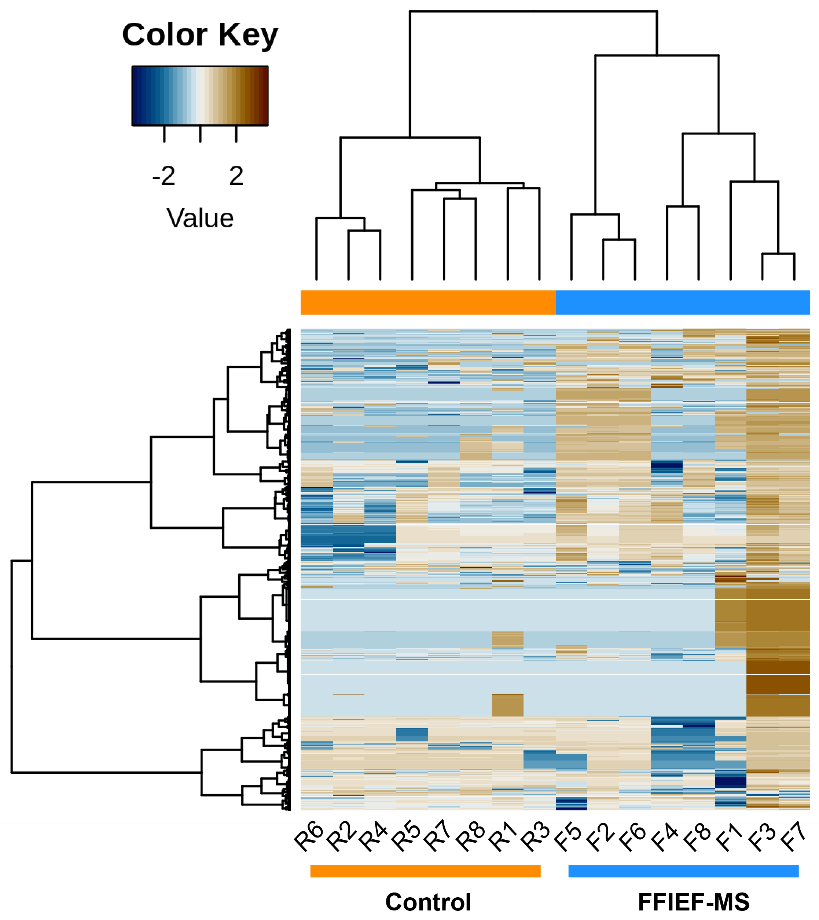


Figure S4. Hierarchically clustered heatmap of bacterial function from microbiome sample that with/without FFIEF (R1-R8, eight 8 replicates that without FFIEF, Control. F1-F8, eight 8 FFIEF fractions, FFIEF-MS). Each row represents a GO term.


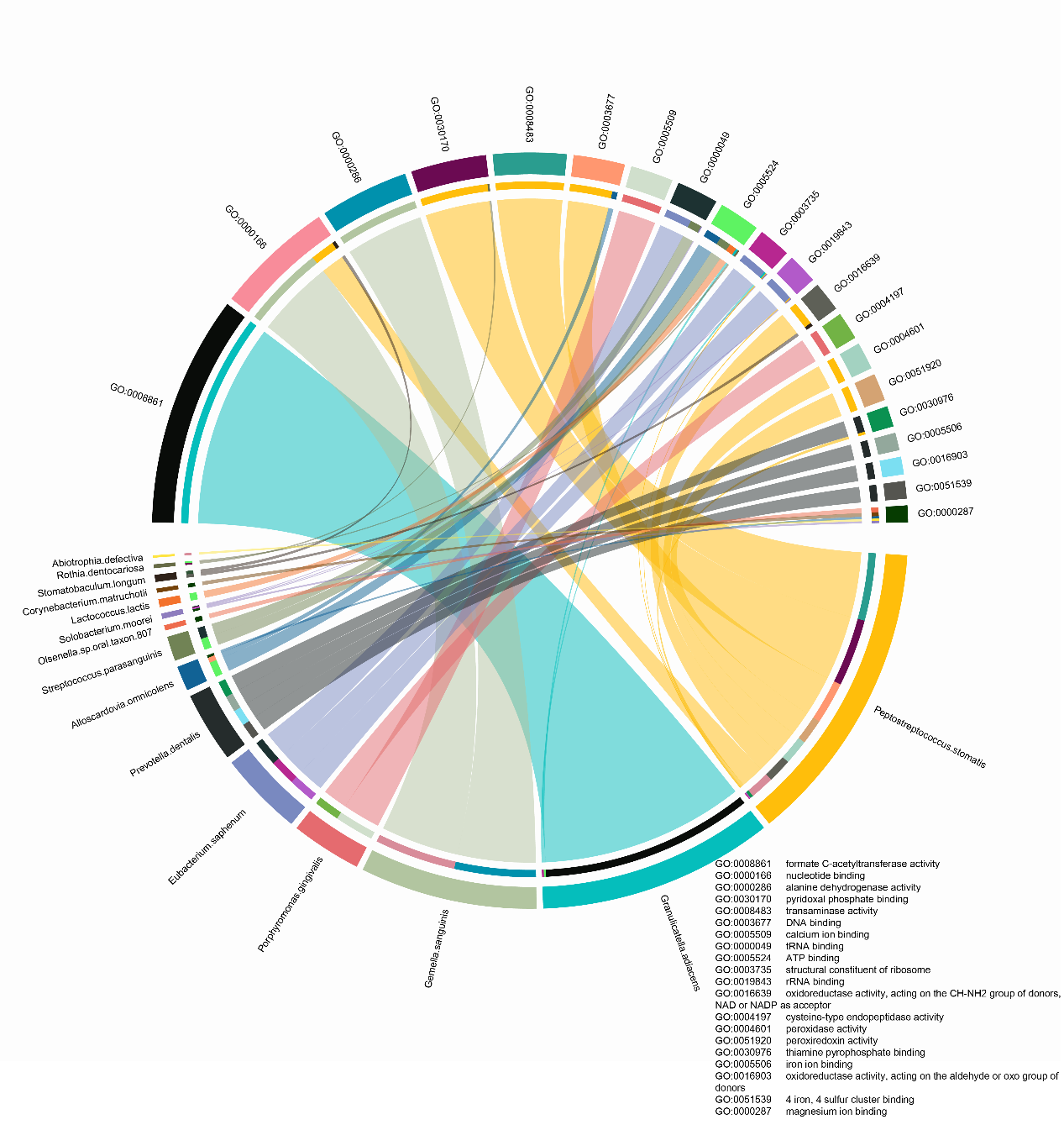


Figure S5. Taxonomy-function integration of the significantly enriched bacterial species by FFIEF and their corresponding functions (top 20 abundant molecular functions). The thickness of each curve corresponds to its relative abundance.


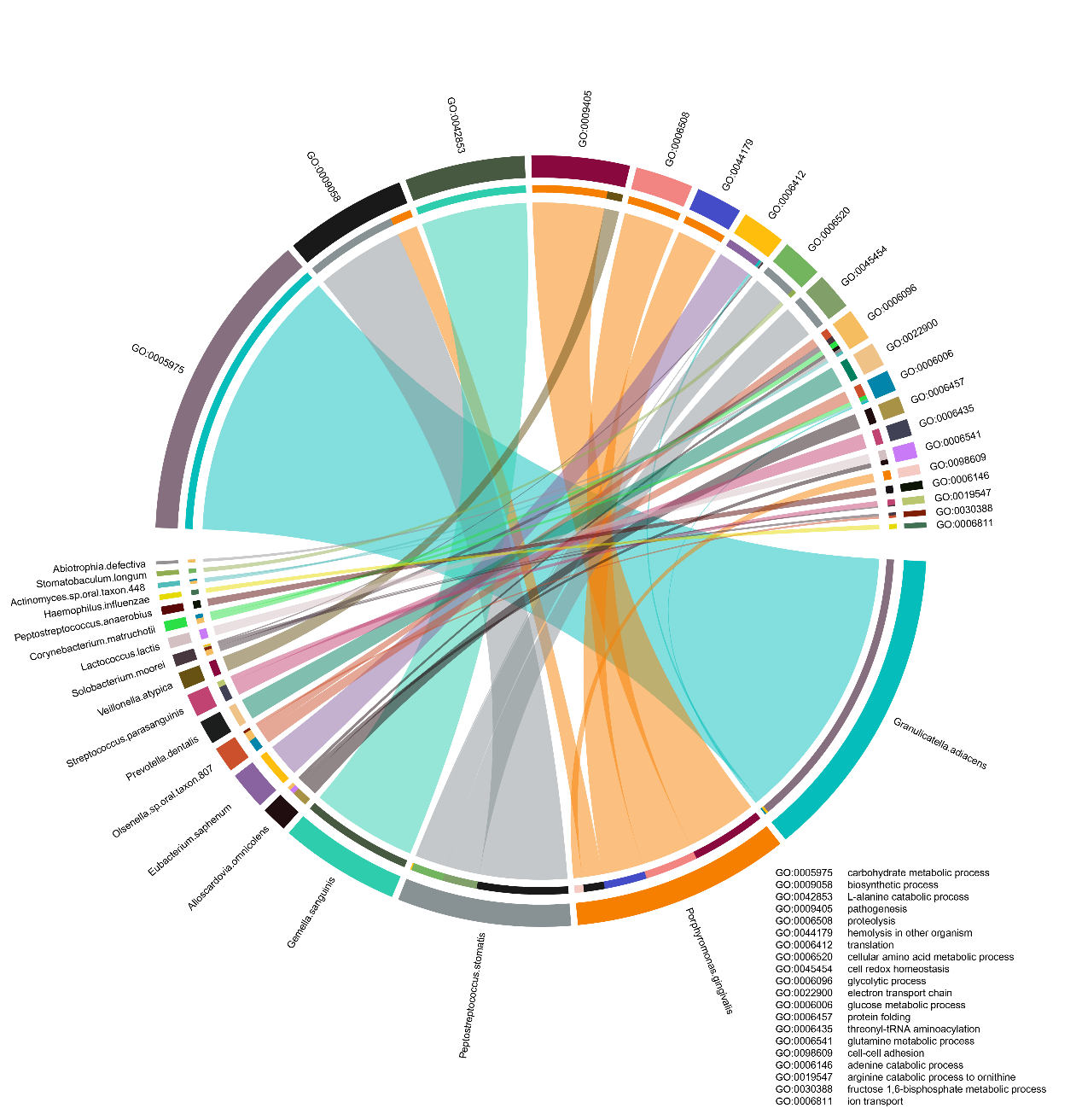


Figure S6. Taxonomy-function integration of the significantly enriched bacterial species by FFIEF and their corresponding functions (top 20 abundant biological processes). The thickness of each curve corresponds to its relative abundance.


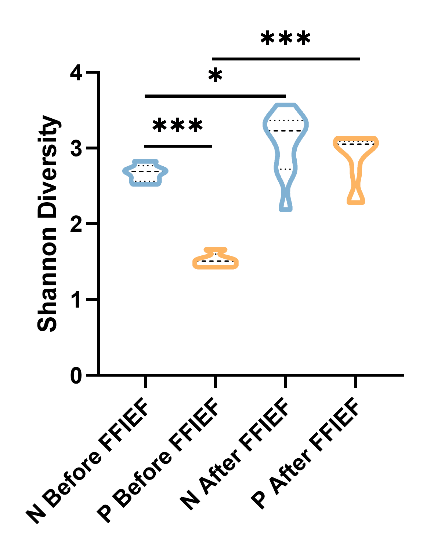


Figure S7. The Shannon diversity of microbiome samples in lung cancer (P) and healthy group (N) that before/after FFIEF. *p < 0.05, **p < 0.01, ***p < 0.001, Wilcoxon rank sum test.


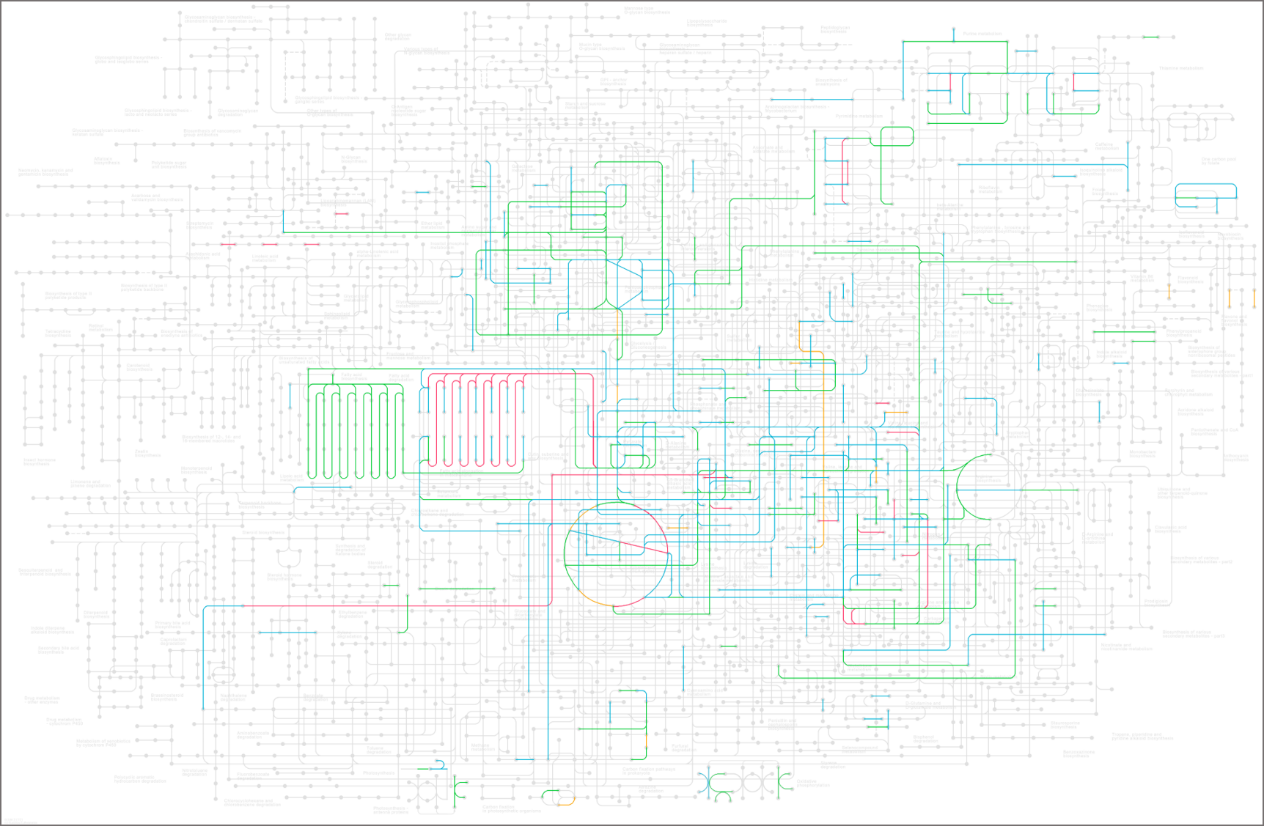


Figure S8. The metabolic pathway constructed by identified bacterial proteins (colored lines). The green lines and red lines represented the significantly enriched pathways in the healthy group and lung cancer group, respectively. The blue lines were fundamental pathways that without significant change, and the orange lines represented pathways enriched in both groups.

Figure S9. The fatty acid metabolism pathway constructed by identified bacterial proteins (colored lines). The green lines represented the pathways that were significantly decreased in lung cancer group. The red lines represented the significantly increased pathways in lung cancer group. The blue lines represented the unchanged pathways between lung cancer and healthy group.


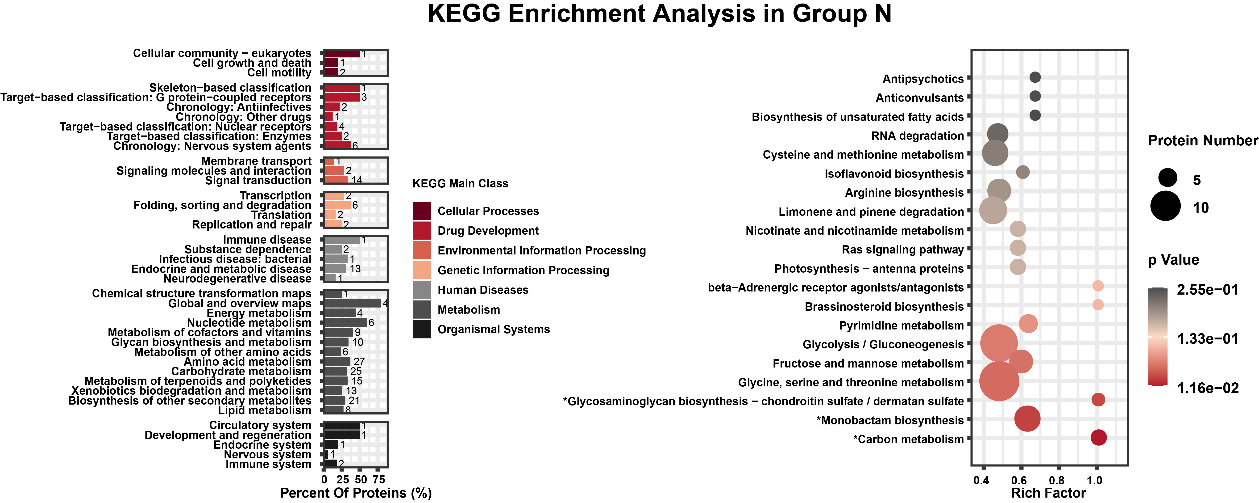


Figure S10. KEGG enrichment analysis in lung cancer group (group N). The left bar plot shows the enriched pathways at the secondary level. Numbers on the graph represent the identified proteins belonging to the respective group. Percent of proteins means the proportion of identified proteins compared with inputted background proteins. The right bubble plot shows the enriched pathways at the tertiary level. Rich factor represents the enrichment level.

**Supplemental tables**

Table S1. Demographic information of enrolled lung cancer patients and healthy controls

|  |  | **Method development phase** | **Method Application phase** | | **qPCR validation phase** | |
| --- | --- | --- | --- | --- | --- | --- |
| **Demographic variable** | **Characteristics** | **Healthy control (n = 10)** | **Lung cancer (n = 16)** | **Healthy control (n =18)** | **Lung cancer (n = 24)** | **Healthy control (n =24)** |
| Age | Mean±SD | 64±8 | 55±4 | 61±6 | 52±3 | 60±5 |
| Gender | Male | 5 | 10 | 11 | 13 | 15 |
|  | Female | 5 | 6 | 7 | 11 | 9 |
|  | Total | 10 | 16 | 18 | 24 | 24 |
| Smoking | Yes | 3 | 10 | 6 | 14 | 8 |
| Ethincity | Asian | 10 | 16 | 18 | 24 | 24 |

Table S2. Taxonomy composition (species level) of microbiome samples that with or without FFIEF fractionation

| **Phylum** | **Species** | **F1** | **F2** | **F3** | **F4** | **F5** | **F6** | **F7** | **F8** | **FFIEF-MS** | **Control method** | **R1** | **R2** | **R3** | **R4** | **R5** | **R6** | **R7** | **R8** |
| --- | --- | --- | --- | --- | --- | --- | --- | --- | --- | --- | --- | --- | --- | --- | --- | --- | --- | --- | --- |
| Actinobacteria | Actinomyces graevenitzii | 0.00 | 2.55 | 1.86 | 0.42 | 2.39 | 2.49 | 1.62 | 2.29 | 1.77 | 1.42 | 1.60 | 1.92 | 1.63 | 0.27 | 1.59 | 1.92 | 2.34 | 1.05 |
| Actinobacteria | Actinomyces odontolyticus | 0.96 | 0.00 | 0.00 | 0.00 | 0.00 | 0.00 | 0.00 | 0.00 | 0.65 | 0.47 | 0.64 | 0.47 | 0.00 | 0.45 | 0.00 | 0.00 | 0.47 | 0.56 |
| Actinobacteria | Actinomyces sp. oral taxon 181 | 0.00 | 0.56 | 0.20 | 0.00 | 0.41 | 0.00 | 0.00 | 0.66 | 0.42 | 0.27 | 0.38 | 0.15 | 0.32 | 0.30 | 0.31 | 0.25 | 0.35 | 0.32 |
| Actinobacteria | Actinomyces sp. oral taxon 414 | 1.43 | 1.53 | 2.28 | 0.00 | 1.06 | 1.54 | 1.97 | 1.10 | 1.35 | 1.73 | 2.63 | 1.89 | 2.32 | 1.89 | 2.39 | 1.75 | 0.72 | 1.61 |
| Actinobacteria | Actinomyces sp. oral taxon 448 | 0.00 | 0.16 | 0.18 | 0.00 | 0.11 | 0.24 | 0.00 | 0.00 | 0.17 | 0.00 | 0.00 | 0.00 | 0.00 | 0.00 | 0.00 | 0.00 | 0.00 | 0.00 |
| Actinobacteria | Actinomyces viscosus | 0.00 | 0.46 | 0.00 | 0.00 | 0.00 | 0.18 | 0.00 | 0.00 | 0.31 | 0.31 | 0.25 | 0.56 | 0.00 | 0.39 | 0.48 | 0.28 | 0.18 | 0.19 |
| Actinobacteria | Alloscardovia omnicolens | 1.59 | 0.00 | 0.00 | 1.88 | 0.10 | 0.00 | 0.00 | 1.31 | 0.73 | 0.24 | 0.34 | 0.00 | 0.00 | 0.00 | 0.00 | 0.00 | 0.00 | 0.00 |
| Actinobacteria | Corynebacterium matruchotii | 0.99 | 0.22 | 2.02 | 0.00 | 0.60 | 1.41 | 0.39 | 0.99 | 0.82 | 0.52 | 0.00 | 0.35 | 0.00 | 1.52 | 0.22 | 0.00 | 0.35 | 0.24 |
| Actinobacteria | Olsenella sp. oral taxon 807 | 0.91 | 0.14 | 0.00 | 0.42 | 0.00 | 0.00 | 0.00 | 0.37 | 0.30 | 0.00 | 0.00 | 0.00 | 0.00 | 0.00 | 0.00 | 0.00 | 0.00 | 0.00 |
| Actinobacteria | Parascardovia denticolens | 0.00 | 0.11 | 1.27 | 0.00 | 0.79 | 0.19 | 0.95 | 0.40 | 0.56 | 0.72 | 0.98 | 0.84 | 0.00 | 0.66 | 0.94 | 0.00 | 0.69 | 0.74 |
| Actinobacteria | Rothia dentocariosa | 0.00 | 0.13 | 0.00 | 0.00 | 0.00 | 0.12 | 0.00 | 0.00 | 0.12 | 0.07 | 0.00 | 0.00 | 0.00 | 0.00 | 0.08 | 0.00 | 0.00 | 0.00 |
| Actinobacteria | Rothia mucilaginosa | 33.11 | 39.78 | 28.51 | 39.87 | 25.98 | 29.60 | 32.85 | 31.42 | 26.14 | 18.80 | 25.24 | 19.96 | 18.18 | 21.12 | 19.72 | 16.11 | 21.05 | 22.34 |
| Actinobacteria | Varibaculum cambriense | 0.00 | 5.39 | 2.75 | 0.00 | 2.70 | 2.81 | 3.21 | 1.60 | 2.86 | 3.71 | 4.26 | 3.38 | 3.64 | 3.25 | 5.29 | 4.56 | 3.99 | 3.83 |
| Bacteroidetes | Alloprevotella tannerae | 0.99 | 0.20 | 0.93 | 1.52 | 0.68 | 0.74 | 1.00 | 1.26 | 0.69 | 0.68 | 0.53 | 0.87 | 0.70 | 0.37 | 0.95 | 0.63 | 1.18 | 0.63 |
| Bacteroidetes | Porphyromonas gingivalis | 0.00 | 1.69 | 1.00 | 0.86 | 0.81 | 1.67 | 0.36 | 4.03 | 1.23 | 0.69 | 1.51 | 0.85 | 1.08 | 0.63 | 0.52 | 0.71 | 0.47 | 0.39 |
| Bacteroidetes | Prevotella dentalis | 0.00 | 0.50 | 1.62 | 0.00 | 0.00 | 1.52 | 0.26 | 0.00 | 0.88 | 0.07 | 0.00 | 0.00 | 0.00 | 0.00 | 0.00 | 0.00 | 0.00 | 0.07 |
| Bacteroidetes | Prevotella intermedia | 0.00 | 0.64 | 0.94 | 1.24 | 0.33 | 0.32 | 0.85 | 1.79 | 0.66 | 0.62 | 1.08 | 0.70 | 0.73 | 0.32 | 0.85 | 0.64 | 0.84 | 0.38 |
| Bacteroidetes | Prevotella melaninogenica | 0.00 | 0.52 | 1.35 | 0.00 | 1.50 | 0.19 | 0.30 | 1.24 | 0.80 | 3.01 | 6.97 | 1.31 | 5.90 | 4.45 | 1.29 | 4.62 | 1.07 | 1.05 |
| Bacteroidetes | Prevotella nigrescens | 1.40 | 0.00 | 0.90 | 0.00 | 0.41 | 0.62 | 1.45 | 2.99 | 1.01 | 1.14 | 0.89 | 1.75 | 1.19 | 1.81 | 1.53 | 0.00 | 0.64 | 0.86 |
| Bacteroidetes | Prevotella pallens | 3.58 | 5.32 | 4.05 | 10.78 | 3.91 | 2.89 | 2.85 | 10.74 | 4.11 | 5.82 | 8.91 | 7.30 | 6.75 | 2.56 | 3.44 | 8.83 | 6.99 | 5.65 |
| Bacteroidetes | Prevotella saccharolytica | 0.00 | 0.25 | 0.00 | 0.00 | 0.17 | 0.23 | 0.00 | 0.00 | 0.22 | 0.31 | 0.00 | 0.00 | 0.00 | 0.00 | 0.00 | 0.29 | 0.00 | 0.00 |
| Bacteroidetes | Prevotella sp. C561 | 0.00 | 0.10 | 0.00 | 0.00 | 0.00 | 0.30 | 4.54 | 0.13 | 0.98 | 0.92 | 0.23 | 0.00 | 0.00 | 0.00 | 0.18 | 1.94 | 0.15 | 2.08 |
| Bacteroidetes | Prevotella sp. oral taxon 306 | 1.93 | 0.77 | 0.76 | 0.00 | 0.96 | 1.40 | 0.94 | 0.00 | 0.99 | 0.87 | 1.88 | 1.60 | 0.44 | 0.47 | 0.00 | 0.50 | 0.45 | 1.37 |
| Bacteroidetes | Tannerella forsythia | 0.00 | 0.22 | 0.00 | 0.00 | 0.00 | 0.00 | 0.28 | 0.51 | 0.27 | 0.26 | 0.00 | 0.25 | 0.00 | 0.27 | 0.00 | 0.00 | 0.00 | 0.00 |
| Bacteroidetes | Tannerella sp. oral taxon HOT-286 | 1.41 | 0.14 | 0.19 | 0.00 | 0.38 | 0.16 | 0.00 | 0.18 | 0.33 | 0.23 | 0.33 | 0.21 | 0.00 | 0.00 | 0.00 | 0.27 | 0.22 | 0.00 |
| Firmicutes | [Eubacterium] brachy | 0.00 | 0.00 | 0.00 | 0.00 | 0.06 | 0.00 | 0.00 | 0.00 | 0.07 | 0.61 | 0.00 | 0.70 | 0.65 | 0.51 | 0.66 | 0.62 | 0.79 | 0.51 |
| Firmicutes | [Eubacterium] saphenum | 1.39 | 1.06 | 0.99 | 7.40 | 0.39 | 1.33 | 0.00 | 2.87 | 1.38 | 0.76 | 1.62 | 0.56 | 0.91 | 0.88 | 0.00 | 0.72 | 0.40 | 0.00 |
| Firmicutes | Abiotrophia defectiva | 0.00 | 0.11 | 0.00 | 0.47 | 0.00 | 0.00 | 0.00 | 0.00 | 0.15 | 0.20 | 0.00 | 0.00 | 0.22 | 0.00 | 0.00 | 0.19 | 0.00 | 0.00 |
| Firmicutes | Erysipelothrix rhusiopathiae | 0.00 | 12.88 | 24.32 | 0.00 | 36.12 | 14.67 | 16.30 | 0.00 | 20.63 | 28.45 | 2.45 | 32.05 | 35.19 | 37.85 | 34.48 | 30.98 | 29.07 | 36.31 |
| Firmicutes | Filifactor alocis | 0.59 | 0.96 | 0.93 | 0.88 | 0.10 | 1.14 | 1.41 | 1.21 | 0.71 | 0.62 | 1.04 | 1.12 | 0.30 | 0.29 | 0.27 | 0.62 | 1.13 | 0.69 |
| Firmicutes | Gemella haemolysans | 0.39 | 0.00 | 0.00 | 4.25 | 0.00 | 0.00 | 0.00 | 0.25 | 0.73 | 0.21 | 0.22 | 0.19 | 0.23 | 0.39 | 0.00 | 0.15 | 0.16 | 0.00 |
| Firmicutes | Gemella sanguinis | 0.61 | 4.59 | 7.18 | 0.68 | 2.66 | 8.97 | 7.27 | 1.37 | 3.70 | 1.35 | 1.81 | 1.31 | 1.14 | 1.40 | 1.56 | 0.96 | 1.97 | 1.64 |
| Firmicutes | Granulicatella adiacens | 21.20 | 0.11 | 0.19 | 0.00 | 0.20 | 0.28 | 0.16 | 0.46 | 2.22 | 0.12 | 0.13 | 0.00 | 0.22 | 0.07 | 0.15 | 0.00 | 0.00 | 0.00 |
| Firmicutes | Granulicatella elegans | 0.00 | 0.13 | 0.16 | 0.00 | 0.14 | 0.13 | 0.18 | 0.00 | 0.14 | 0.00 | 0.00 | 0.00 | 0.00 | 0.00 | 0.00 | 0.00 | 0.00 | 0.00 |
| Firmicutes | Lachnoanaerobaculum saburreum | 0.00 | 0.16 | 0.07 | 0.00 | 0.12 | 0.00 | 0.09 | 0.00 | 0.11 | 0.16 | 0.00 | 0.17 | 0.00 | 0.00 | 0.00 | 0.00 | 0.00 | 0.00 |
| Firmicutes | Lactococcus lactis | 0.00 | 0.92 | 1.34 | 9.34 | 4.06 | 10.17 | 1.87 | 5.14 | 3.65 | 1.78 | 6.37 | 0.51 | 0.53 | 0.00 | 0.00 | 0.31 | 4.65 | 0.54 |
| Firmicutes | Megasphaera micronuciformis | 0.43 | 1.48 | 1.16 | 0.00 | 2.26 | 1.12 | 0.39 | 0.70 | 1.05 | 2.86 | 3.60 | 3.23 | 2.92 | 2.75 | 3.46 | 2.75 | 3.21 | 2.95 |
| Firmicutes | Oribacterium sinus | 0.00 | 0.79 | 0.37 | 0.00 | 0.13 | 0.16 | 0.26 | 1.50 | 0.45 | 0.00 | 0.00 | 0.00 | 0.00 | 0.00 | 0.00 | 0.00 | 0.00 | 0.00 |
| Firmicutes | Peptostreptococcus anaerobius | 0.00 | 0.51 | 0.00 | 0.00 | 0.00 | 0.00 | 0.00 | 0.16 | 0.31 | 0.00 | 0.00 | 0.00 | 0.00 | 0.00 | 0.00 | 0.00 | 0.00 | 0.00 |
| Firmicutes | Peptostreptococcus stomatis | 14.45 | 3.42 | 2.37 | 1.50 | 2.07 | 1.88 | 3.55 | 5.54 | 3.34 | 3.19 | 5.59 | 2.87 | 3.58 | 4.48 | 4.46 | 1.91 | 2.98 | 2.47 |
| Firmicutes | Solobacterium moorei | 0.00 | 0.13 | 0.20 | 0.00 | 0.00 | 0.00 | 0.00 | 0.88 | 0.31 | 0.14 | 0.00 | 0.12 | 0.00 | 0.00 | 0.00 | 0.22 | 0.07 | 0.00 |
| Firmicutes | Stomatobaculum longum | 0.00 | 2.52 | 0.64 | 0.89 | 0.37 | 0.66 | 0.54 | 0.93 | 0.79 | 0.14 | 0.00 | 0.14 | 0.00 | 0.00 | 0.00 | 0.00 | 0.00 | 0.00 |
| Firmicutes | Streptococcus cristatus | 0.26 | 1.23 | 0.00 | 0.00 | 0.00 | 0.36 | 0.00 | 0.58 | 0.54 | 0.95 | 2.27 | 0.00 | 0.27 | 0.00 | 0.21 | 2.17 | 0.00 | 0.43 |
| Firmicutes | Streptococcus infantis | 0.69 | 1.07 | 0.43 | 1.51 | 0.78 | 1.70 | 1.39 | 2.53 | 0.99 | 1.04 | 1.61 | 1.09 | 1.13 | 0.97 | 0.89 | 0.95 | 1.19 | 1.29 |
| Firmicutes | Streptococcus mitis | 1.37 | 1.17 | 2.22 | 5.05 | 0.51 | 1.57 | 1.96 | 2.44 | 1.42 | 2.85 | 2.03 | 3.91 | 3.82 | 1.37 | 4.60 | 1.94 | 3.94 | 3.19 |
| Firmicutes | Streptococcus parasanguinis | 0.00 | 0.40 | 0.00 | 2.92 | 0.12 | 0.21 | 0.92 | 0.47 | 0.49 | 0.14 | 0.00 | 0.00 | 0.17 | 0.12 | 0.00 | 0.00 | 0.00 | 0.00 |
| Firmicutes | Streptococcus pneumoniae | 0.00 | 0.00 | 0.00 | 0.00 | 0.00 | 0.00 | 0.00 | 0.10 | 0.07 | 0.73 | 0.00 | 0.00 | 0.76 | 0.56 | 0.99 | 0.00 | 0.00 | 0.84 |
| Firmicutes | Veillonella atypica | 0.00 | 0.15 | 0.09 | 0.81 | 0.08 | 0.28 | 0.00 | 1.33 | 0.31 | 0.00 | 0.00 | 0.00 | 0.00 | 0.00 | 0.00 | 0.00 | 0.00 | 0.00 |
| Firmicutes | Veillonella dispar | 1.18 | 0.00 | 0.50 | 0.00 | 0.81 | 1.04 | 0.00 | 0.45 | 0.71 | 1.61 | 2.40 | 0.00 | 0.00 | 0.00 | 0.00 | 1.13 | 0.00 | 1.93 |
| Fusobacteria | Fusobacterium periodonticum | 0.00 | 0.17 | 0.19 | 0.77 | 0.00 | 0.00 | 0.00 | 0.00 | 0.21 | 0.24 | 0.32 | 0.21 | 0.31 | 0.23 | 0.28 | 0.19 | 0.31 | 0.27 |
| Fusobacteria | Leptotrichia sp. oral taxon 212 | 0.00 | 0.28 | 0.42 | 0.00 | 0.00 | 0.39 | 0.97 | 0.86 | 0.47 | 0.21 | 0.24 | 0.25 | 0.00 | 0.20 | 0.00 | 0.00 | 0.00 | 0.00 |
| Fusobacteria | Leptotrichia sp. oral taxon 215 | 0.00 | 0.28 | 0.70 | 0.00 | 0.00 | 0.88 | 0.78 | 0.13 | 0.48 | 0.82 | 1.77 | 1.31 | 1.35 | 0.21 | 0.12 | 0.00 | 0.90 | 0.00 |
| Fusobacteria | Leptotrichia sp. oral taxon 847 | 0.00 | 0.24 | 0.00 | 0.00 | 0.00 | 0.00 | 0.24 | 0.00 | 0.21 | 0.99 | 0.64 | 0.00 | 0.00 | 0.27 | 1.58 | 1.47 | 0.00 | 1.33 |
| Fusobacteria | Leptotrichia wadei | 3.14 | 2.46 | 2.67 | 1.61 | 3.78 | 2.09 | 1.58 | 0.97 | 1.99 | 1.72 | 1.64 | 1.28 | 1.29 | 3.52 | 1.64 | 3.60 | 0.74 | 0.80 |
| Proteobacteria | Cardiobacterium hominis | 0.40 | 0.00 | 0.00 | 0.00 | 0.00 | 0.00 | 0.18 | 0.00 | 0.20 | 0.10 | 0.00 | 0.00 | 0.00 | 0.08 | 0.14 | 0.00 | 0.00 | 0.00 |
| Proteobacteria | Haemophilus influenzae | 0.00 | 0.00 | 0.00 | 0.00 | 0.21 | 0.00 | 0.00 | 0.00 | 0.25 | 0.00 | 0.00 | 0.00 | 0.00 | 0.00 | 0.00 | 0.00 | 0.00 | 0.00 |
| Proteobacteria | Lautropia mirabilis | 0.00 | 0.00 | 0.00 | 0.00 | 0.00 | 0.00 | 0.00 | 0.88 | 0.64 | 0.23 | 0.00 | 0.00 | 0.00 | 0.00 | 0.00 | 0.22 | 0.00 | 0.00 |
| Proteobacteria | Neisseria flavescens | 0.00 | 0.00 | 0.12 | 0.00 | 0.10 | 0.00 | 0.20 | 0.35 | 0.16 | 1.56 | 2.10 | 1.39 | 2.11 | 1.50 | 1.97 | 1.34 | 1.69 | 0.00 |
| Proteobacteria | Neisseria mucosa | 5.59 | 1.40 | 1.94 | 4.91 | 1.62 | 2.35 | 5.93 | 4.65 | 2.60 | 2.16 | 3.51 | 1.83 | 0.00 | 0.16 | 2.80 | 3.29 | 3.62 | 1.47 |
| Proteobacteria | Neisseria sp. oral taxon 014 | 0.00 | 0.00 | 0.00 | 0.00 | 0.00 | 0.00 | 0.00 | 0.22 | 0.16 | 1.19 | 0.00 | 1.40 | 0.00 | 1.41 | 0.00 | 0.99 | 1.03 | 0.00 |

Table S3. The significantly enriched species after FFIEF

| **Exp.** | **No.** | **Species** | **BEFORE_FFIEF** | **AFTER_FFIEF** | **p-values (corrected)** | **Effect size** | **95.0% lower CI** | **95.0% upper CI** | **Fold_Change** |
| --- | --- | --- | --- | --- | --- | --- | --- | --- | --- |
| EXP. 1 | 1 | [Eubacterium] saphenum | 0.61 | 7.40 | 0.00 | 6.79 | -0.52 | 14.11 | 12.07 |
|  | 2 | Abiotrophia defectiva | 0.05 | 0.47 | 0.00 | 0.42 | -2.96 | 3.79 | 8.70 |
|  | 3 | Actinomyces sp. oral taxon 448 | 0.00 | 0.24 | 0.00 | 0.10 | 0.03 | 0.16 | 100.00 |
|  | 4 | Alloscardovia omnicolens | 0.03 | 1.31 | 0.02 | 0.57 | 0.05 | 1.10 | 42.23 |
|  | 5 | Cardiobacterium hominis | 0.03 | 0.40 | 0.00 | 0.37 | -2.86 | 3.61 | 14.76 |
|  | 6 | Corynebacterium matruchotii | 0.35 | 2.02 | 0.04 | 0.44 | -0.05 | 0.93 | 5.76 |
|  | 7 | Gemella sanguinis | 1.42 | 8.97 | 0.01 | 2.73 | 0.63 | 4.83 | 6.30 |
|  | 8 | Granulicatella adiacens | 0.07 | 21.20 | 0.00 | 21.14 | 11.15 | 31.12 | 318.34 |
|  | 9 | Granulicatella elegans | 0.00 | 0.18 | 0.00 | 0.10 | 0.05 | 0.15 | 100.00 |
|  | 10 | Haemophilus influenzae | 0.00 | 0.21 | 0.00 | 0.21 | -2.64 | 3.06 | 100.00 |
|  | 11 | Lachnoanaerobaculum saburreum | 0.02 | 0.16 | 0.00 | 0.14 | -2.66 | 2.93 | 7.28 |
|  | 12 | Lactococcus lactis | 1.44 | 10.17 | 0.00 | 8.73 | 0.40 | 17.06 | 7.05 |
|  | 13 | Leptotrichia sp. oral taxon 212 | 0.08 | 0.42 | 0.01 | 0.29 | 0.05 | 0.54 | 5.10 |
|  | 14 | Olsenella sp. oral taxon 807 | 0.00 | 0.42 | 0.02 | 0.23 | 0.03 | 0.44 | 100.00 |
|  | 15 | Oribacterium sinus | 0.00 | 0.41 | 0.01 | 0.41 | 0.08 | 0.73 | 100.00 |
|  | 16 | Peptostreptococcus anaerobius | 0.00 | 0.16 | 0.00 | 0.16 | -2.59 | 2.92 | 100.00 |
|  | 17 | Peptostreptococcus stomatis | 3.44 | 14.45 | 0.00 | 11.01 | 1.29 | 20.74 | 4.21 |
|  | 18 | Porphyromonas gingivalis | 0.75 | 4.03 | 0.03 | 3.28 | -2.88 | 9.45 | 5.40 |
|  | 19 | Prevotella dentalis | 0.01 | 0.26 | 0.00 | 0.25 | -2.73 | 3.23 | 26.34 |
|  | 20 | Prevotella saccharolytica | 0.04 | 0.25 | 0.00 | 0.21 | -2.81 | 3.24 | 6.07 |
|  | 21 | Rothia dentocariosa | 0.01 | 0.13 | 0.00 | 0.12 | -2.57 | 2.81 | 14.70 |
|  | 22 | Solobacterium moorei | 0.06 | 0.20 | 0.00 | 0.14 | -2.81 | 3.09 | 3.50 |
|  | 23 | Stomatobaculum longum | 0.02 | 0.37 | 0.00 | 0.35 | -2.83 | 3.54 | 20.11 |
|  | 24 | Streptococcus parasanguinis | 0.04 | 0.47 | 0.00 | 0.43 | -2.92 | 3.78 | 12.35 |
|  | 25 | Tannerella forsythia | 0.07 | 0.28 | 0.00 | 0.21 | -2.91 | 3.33 | 4.08 |
|  | 26 | Tannerella sp. oral taxon HOT-286 | 0.13 | 1.41 | 0.00 | 1.29 | -3.09 | 5.66 | 11.17 |
|  | 27 | Veillonella atypica | 0.00 | 1.33 | 0.02 | 0.33 | 0.03 | 0.64 | 100.00 |
| Exp. 2 | 1 | Aggregatibacter segnis | 0.00 | 0.09 | 0.04 | 0.09 | -0.02 | 0.19 | 100.00 |
|  | 2 | Alloprevotella tannerae | 0.05 | 0.56 | 0.01 | 0.50 | 0.11 | 0.90 | 10.54 |
|  | 3 | Campylobacter rectus | 0.00 | 0.20 | 0.01 | 0.20 | 0.04 | 0.37 | 100.00 |
|  | 4 | Cardiobacterium hominis | 0.00 | 0.22 | 0.01 | 0.22 | 0.06 | 0.37 | 100.00 |
|  | 5 | Delftia acidovorans | 0.00 | 1.10 | 0.03 | 1.10 | -0.04 | 2.24 | 100.00 |
|  | 6 | Filifactor alocis | 0.59 | 1.54 | 0.00 | 0.95 | 0.38 | 1.52 | 2.62 |
|  | 7 | Fusobacterium nucleatum | 0.00 | 0.02 | 0.02 | 0.02 | 0.00 | 0.04 | 100.00 |
|  | 8 | Haemophilus parainfluenzae | 0.00 | 0.08 | 0.02 | 0.08 | 0.01 | 0.15 | 100.00 |
|  | 9 | Neisseria elongata | 0.00 | 0.59 | 0.02 | 0.59 | 0.01 | 1.17 | 100.00 |
|  | 10 | Neisseria flavescens | 0.21 | 2.73 | 0.01 | 2.52 | 0.54 | 4.50 | 13.05 |
|  | 11 | Neisseria sp. oral taxon 014 | 0.64 | 5.16 | 0.00 | 4.52 | 1.78 | 7.25 | 8.03 |
|  | 12 | Olsenella sp. oral taxon 807 | 0.02 | 0.19 | 0.02 | 0.17 | 0.01 | 0.34 | 12.14 |
|  | 13 | Peptostreptococcus stomatis | 0.23 | 0.70 | 0.02 | 0.47 | 0.05 | 0.89 | 3.03 |
|  | 14 | Porphyromonas endodontalis | 0.11 | 0.40 | 0.04 | 0.30 | -0.05 | 0.64 | 3.69 |
|  | 15 | Porphyromonas gingivalis | 1.42 | 3.42 | 0.02 | 1.99 | 0.15 | 3.83 | 2.40 |
|  | 16 | Prevotella histicola | 0.02 | 0.45 | 0.01 | 0.43 | 0.11 | 0.76 | 24.49 |
|  | 17 | Prevotella sp. oral taxon 306 | 0.00 | 0.70 | 0.04 | 0.70 | -0.12 | 1.53 | 100.00 |
|  | 18 | Ralstonia pickettii | 0.00 | 1.31 | 0.02 | 1.31 | 0.06 | 2.55 | 100.00 |
|  | 19 | Rothia mucilaginosa | 0.27 | 2.33 | 0.02 | 2.07 | 0.10 | 4.03 | 8.77 |
|  | 20 | Selenomonas noxia | 0.00 | 0.21 | 0.03 | 0.21 | -0.01 | 0.42 | 100.00 |
|  | 21 | Selenomonas sp. oral taxon 126 | 0.00 | 0.47 | 0.01 | 0.47 | 0.09 | 0.84 | 100.00 |
|  | 22 | Selenomonas sp. oral taxon 478 | 0.00 | 0.37 | 0.01 | 0.37 | 0.09 | 0.66 | 100.00 |
|  | 23 | Solobacterium moorei | 0.00 | 0.23 | 0.02 | 0.23 | 0.02 | 0.45 | 100.00 |
|  | 24 | Stomatobaculum longum | 0.02 | 0.68 | 0.02 | 0.66 | 0.05 | 1.27 | 30.96 |
|  | 25 | Veillonella atypica | 0.00 | 0.89 | 0.03 | 0.89 | -0.09 | 1.88 | 100.00 |
| Exp. 3 | 1 | Aggregatibacter segnis | 0.00 | 0.11 | 0.03 | 0.11 | 0.00 | 0.23 | 100.00 |
|  | 2 | Aggregatibacter sp. oral taxon 458 | 0.03 | 1.77 | 0.00 | 1.74 | 0.75 | 2.73 | 67.17 |
|  | 3 | Alloprevotella rava | 0.00 | 0.20 | 0.01 | 0.20 | 0.05 | 0.35 | 100.00 |
|  | 4 | Campylobacter showae | 0.00 | 0.82 | 0.00 | 0.82 | 0.62 | 1.01 | 100.00 |
|  | 5 | Capnocytophaga sp. oral taxon 329 | 0.00 | 0.12 | 0.01 | 0.12 | 0.02 | 0.21 | 100.00 |
|  | 6 | Cardiobacterium hominis | 0.07 | 0.85 | 0.00 | 0.78 | 0.38 | 1.18 | 11.97 |
|  | 7 | Cardiobacterium valvarum | 0.00 | 0.40 | 0.00 | 0.40 | 0.15 | 0.64 | 100.00 |
|  | 8 | Catonella morbi | 0.00 | 0.13 | 0.02 | 0.13 | 0.01 | 0.24 | 100.00 |
|  | 9 | Dialister invisus | 0.00 | 0.19 | 0.01 | 0.19 | 0.03 | 0.35 | 100.00 |
|  | 10 | Filifactor alocis | 0.12 | 3.48 | 0.04 | 3.35 | -0.66 | 7.37 | 28.50 |
|  | 11 | Fretibacterium fastidiosum | 0.00 | 2.54 | 0.04 | 2.54 | -0.38 | 5.47 | 100.00 |
|  | 12 | Fusobacterium nucleatum | 0.06 | 8.86 | 0.01 | 8.81 | 1.57 | 16.04 | 160.53 |
|  | 13 | Fusobacterium periodonticum | 0.00 | 0.74 | 0.00 | 0.74 | 0.42 | 1.06 | 100.00 |
|  | 14 | Lautropia mirabilis | 0.96 | 5.61 | 0.00 | 4.65 | 2.43 | 6.88 | 5.86 |
|  | 15 | Leptotrichia buccalis | 0.00 | 1.16 | 0.01 | 1.16 | 0.34 | 1.98 | 100.00 |
|  | 16 | Leptotrichia sp. oral taxon 212 | 0.00 | 0.37 | 0.01 | 0.37 | 0.08 | 0.66 | 100.00 |
|  | 17 | Neisseria cinerea | 0.00 | 0.09 | 0.03 | 0.09 | 0.00 | 0.19 | 100.00 |
|  | 18 | Neisseria elongata | 0.00 | 1.28 | 0.00 | 1.28 | 0.46 | 2.09 | 100.00 |
|  | 19 | Neisseria mucosa | 2.20 | 4.69 | 0.05 | 2.49 | -0.56 | 5.55 | 2.14 |
|  | 20 | Neisseria sicca | 0.00 | 0.18 | 0.03 | 0.18 | -0.01 | 0.38 | 100.00 |
|  | 21 | Neisseria sp. oral taxon 014 | 0.65 | 4.81 | 0.02 | 4.16 | 0.14 | 8.18 | 7.38 |
|  | 22 | Oribacterium parvum | 0.00 | 0.10 | 0.02 | 0.10 | 0.01 | 0.19 | 100.00 |
|  | 23 | Peptostreptococcus stomatis | 0.03 | 0.52 | 0.00 | 0.49 | 0.23 | 0.75 | 16.31 |
|  | 24 | Porphyromonas endodontalis | 0.00 | 0.12 | 0.00 | 0.12 | 0.04 | 0.20 | 100.00 |
|  | 25 | Porphyromonas gingivalis | 0.00 | 3.47 | 0.00 | 3.47 | 1.64 | 5.30 | 100.00 |
|  | 26 | Prevotella intermedia | 0.00 | 0.05 | 0.04 | 0.05 | -0.01 | 0.11 | 100.00 |
|  | 27 | Prevotella melaninogenica | 0.00 | 0.26 | 0.01 | 0.26 | 0.03 | 0.49 | 100.00 |
|  | 28 | Prevotella nigrescens | 0.00 | 0.89 | 0.00 | 0.89 | 0.56 | 1.22 | 100.00 |
|  | 29 | Prevotella pallens | 3.40 | 8.49 | 0.00 | 5.09 | 2.37 | 7.82 | 2.50 |
|  | 30 | Ralstonia pickettii | 0.00 | 0.35 | 0.01 | 0.35 | 0.09 | 0.61 | 100.00 |
|  | 31 | Selenomonas flueggei | 0.00 | 0.99 | 0.01 | 0.99 | 0.26 | 1.72 | 100.00 |
|  | 32 | Streptococcus mitis | 0.07 | 4.37 | 0.01 | 4.30 | 1.29 | 7.31 | 66.56 |
|  | 33 | Tannerella forsythia | 0.00 | 0.71 | 0.00 | 0.71 | 0.29 | 1.13 | 100.00 |
|  | 34 | Tannerella sp. oral taxon HOT-286 | 1.40 | 9.64 | 0.00 | 8.24 | 6.25 | 10.24 | 6.90 |

Table S4. Taxonomy composition (species level) of lung cancer (P) and healthy group (N) in different FFIEF fractions

| **Phylum** | **Species** | **N1** | **P1** | **N2** | **P2** | **N3** | **P3** | **N4** | **P4** | **N5** | **P5** | **N6** | **P6** | **N7** | **P7** | **N8** | **P8** | **N_GROUP** | **P_GROUP** |
| --- | --- | --- | --- | --- | --- | --- | --- | --- | --- | --- | --- | --- | --- | --- | --- | --- | --- | --- | --- |
| Actinobacteria | Actinomyces georgiae | 0.00 | 0.00 | 0.29 | 0.00 | 0.22 | 0.00 | 0.00 | 0.00 | 0.00 | 0.00 | 0.00 | 0.00 | 0.00 | 0.00 | 8.46 | 0.00 | 1.40 | 0.00 |
| Actinobacteria | Actinomyces graevenitzii | 18.66 | 0.37 | 27.69 | 0.22 | 18.02 | 0.47 | 4.85 | 0.24 | 8.85 | 0.29 | 24.03 | 0.88 | 37.71 | 0.16 | 33.37 | 0.00 | 17.15 | 0.29 |
| Actinobacteria | Actinomyces odontolyticus | 1.17 | 0.00 | 1.88 | 0.00 | 1.95 | 0.00 | 0.93 | 0.00 | 0.20 | 0.00 | 1.05 | 0.00 | 4.08 | 0.00 | 3.74 | 0.00 | 1.46 | 0.00 |
| Actinobacteria | Actinomyces sp. oral taxon 172 | 2.24 | 0.00 | 2.48 | 0.00 | 1.56 | 0.00 | 0.00 | 0.00 | 0.76 | 0.00 | 3.08 | 0.00 | 6.43 | 0.00 | 0.00 | 0.00 | 2.35 | 0.00 |
| Actinobacteria | Actinomyces sp. oral taxon 180 | 0.00 | 0.00 | 0.49 | 0.00 | 0.30 | 0.00 | 0.00 | 0.00 | 0.17 | 0.00 | 0.41 | 0.00 | 0.64 | 0.00 | 0.00 | 0.00 | 0.37 | 0.00 |
| Actinobacteria | Actinomyces sp. oral taxon 414 | 0.00 | 0.00 | 0.15 | 0.00 | 0.04 | 0.00 | 0.63 | 0.00 | 0.48 | 0.00 | 0.18 | 0.00 | 0.63 | 0.23 | 0.00 | 0.00 | 0.34 | 0.19 |
| Actinobacteria | Corynebacterium matruchotii | 0.58 | 0.00 | 0.23 | 0.00 | 0.43 | 0.20 | 0.11 | 0.00 | 0.22 | 0.00 | 0.82 | 0.34 | 0.55 | 0.16 | 0.21 | 0.00 | 0.33 | 0.16 |
| Actinobacteria | Olsenella sp. oral taxon 807 | 0.00 | 0.23 | 0.29 | 0.00 | 0.28 | 0.00 | 0.40 | 0.05 | 0.48 | 0.10 | 0.06 | 0.39 | 0.00 | 0.23 | 0.00 | 0.00 | 0.31 | 0.14 |
| Actinobacteria | Rothia dentocariosa | 0.00 | 0.20 | 0.00 | 0.00 | 0.00 | 0.00 | 0.12 | 0.00 | 0.00 | 0.00 | 0.00 | 0.00 | 0.00 | 0.00 | 0.00 | 0.00 | 0.12 | 0.11 |
| Actinobacteria | Rothia mucilaginosa | 0.42 | 1.90 | 3.55 | 2.63 | 3.14 | 2.59 | 6.27 | 2.47 | 4.45 | 0.79 | 0.15 | 3.24 | 0.67 | 2.86 | 0.00 | 1.88 | 2.69 | 1.79 |
| Bacteroidetes | Alloprevotella rava | 0.35 | 0.24 | 0.57 | 0.48 | 1.39 | 0.28 | 2.79 | 0.07 | 1.08 | 0.13 | 1.02 | 0.40 | 0.93 | 0.00 | 0.00 | 0.00 | 1.13 | 0.19 |
| Bacteroidetes | Alloprevotella tannerae | 0.00 | 4.65 | 0.99 | 1.11 | 0.59 | 0.00 | 1.32 | 0.17 | 0.72 | 0.00 | 0.53 | 1.40 | 0.30 | 0.22 | 0.00 | 0.00 | 0.73 | 0.88 |
| Bacteroidetes | Capnocytophaga sp. oral taxon 329 | 0.00 | 0.00 | 0.05 | 0.24 | 0.00 | 0.13 | 0.00 | 0.05 | 0.00 | 0.07 | 0.00 | 0.32 | 0.00 | 0.13 | 0.00 | 0.00 | 0.05 | 0.12 |
| Bacteroidetes | Porphyromonas endodontalis | 0.00 | 0.08 | 0.78 | 0.00 | 0.99 | 0.16 | 0.73 | 0.10 | 0.42 | 0.15 | 0.00 | 0.28 | 0.32 | 0.20 | 0.00 | 0.00 | 0.66 | 0.14 |
| Bacteroidetes | Porphyromonas gingivalis | 1.68 | 1.85 | 4.92 | 2.41 | 6.50 | 2.48 | 2.30 | 2.19 | 5.18 | 2.24 | 2.31 | 6.45 | 3.72 | 2.61 | 0.73 | 7.48 | 3.25 | 2.29 |
| Bacteroidetes | Prevotella histicola | 1.13 | 0.00 | 0.27 | 0.00 | 0.36 | 0.23 | 0.51 | 0.00 | 0.31 | 0.00 | 0.90 | 0.00 | 0.14 | 0.00 | 0.00 | 0.00 | 0.43 | 0.19 |
| Bacteroidetes | Prevotella intermedia | 0.55 | 0.12 | 0.87 | 0.17 | 0.95 | 0.00 | 0.44 | 0.00 | 0.67 | 0.00 | 0.00 | 0.00 | 0.43 | 0.12 | 0.00 | 0.00 | 0.62 | 0.09 |
| Bacteroidetes | Prevotella melaninogenica | 0.27 | 0.51 | 0.72 | 0.68 | 0.43 | 0.50 | 0.14 | 0.28 | 0.13 | 0.00 | 0.26 | 0.00 | 0.34 | 0.12 | 0.00 | 0.00 | 0.30 | 0.33 |
| Bacteroidetes | Prevotella multiformis | 0.00 | 0.00 | 0.00 | 0.00 | 0.00 | 0.24 | 0.74 | 0.00 | 0.14 | 0.00 | 0.00 | 0.00 | 0.00 | 0.00 | 0.00 | 0.00 | 0.45 | 0.20 |
| Bacteroidetes | Prevotella nigrescens | 5.06 | 1.31 | 1.78 | 1.23 | 1.89 | 0.41 | 1.94 | 0.69 | 1.65 | 0.60 | 1.72 | 1.42 | 2.56 | 0.47 | 2.68 | 0.98 | 1.91 | 0.63 |
| Bacteroidetes | Prevotella pallens | 2.17 | 9.82 | 2.99 | 11.10 | 3.08 | 5.99 | 4.71 | 3.37 | 3.26 | 6.07 | 2.73 | 12.60 | 4.36 | 7.64 | 0.00 | 11.34 | 3.09 | 5.71 |
| Bacteroidetes | Prevotella salivae | 0.00 | 0.00 | 0.00 | 0.00 | 0.00 | 0.00 | 0.00 | 0.00 | 0.00 | 0.00 | 0.00 | 0.00 | 0.17 | 0.00 | 0.00 | 0.00 | 0.14 | 0.00 |
| Bacteroidetes | Prevotella sp. C561 | 0.00 | 0.14 | 1.62 | 0.00 | 0.54 | 0.18 | 0.57 | 0.07 | 1.00 | 0.00 | 0.10 | 0.76 | 0.00 | 0.00 | 0.00 | 0.00 | 0.77 | 0.18 |
| Bacteroidetes | Prevotella sp. oral taxon 306 | 0.00 | 0.00 | 0.32 | 0.00 | 2.55 | 0.00 | 1.85 | 0.31 | 0.82 | 0.00 | 0.00 | 0.44 | 0.10 | 0.00 | 0.00 | 0.00 | 1.20 | 0.37 |
| Bacteroidetes | Prevotella sp. oral taxon 473 | 0.07 | 1.18 | 0.55 | 1.46 | 0.72 | 0.39 | 1.56 | 0.43 | 1.26 | 0.82 | 0.00 | 1.41 | 0.55 | 2.25 | 0.00 | 1.16 | 0.79 | 0.80 |
| Bacteroidetes | Tannerella forsythia | 0.00 | 0.67 | 0.24 | 0.41 | 0.16 | 0.21 | 0.32 | 0.82 | 0.47 | 0.63 | 0.00 | 1.83 | 0.25 | 0.76 | 0.00 | 0.34 | 0.29 | 0.57 |
| Bacteroidetes | Tannerella sp. oral taxon HOT-286 | 0.19 | 10.67 | 0.32 | 8.46 | 0.30 | 11.04 | 0.59 | 5.35 | 0.32 | 7.91 | 0.29 | 13.20 | 0.51 | 9.72 | 0.00 | 10.74 | 0.34 | 7.00 |
| Firmicutes | Catonella morbi | 0.69 | 0.21 | 0.58 | 0.00 | 0.47 | 0.10 | 0.11 | 0.07 | 0.34 | 0.09 | 0.62 | 0.43 | 0.16 | 0.11 | 0.46 | 0.00 | 0.35 | 0.12 |
| Firmicutes | Dialister invisus | 0.00 | 0.00 | 0.41 | 0.43 | 0.00 | 0.35 | 1.26 | 0.12 | 0.30 | 0.19 | 0.33 | 0.00 | 0.21 | 0.43 | 1.05 | 0.00 | 0.48 | 0.26 |
| Firmicutes | Filifactor alocis | 1.52 | 0.82 | 0.99 | 2.69 | 1.43 | 1.46 | 1.12 | 1.64 | 2.82 | 0.60 | 0.77 | 4.03 | 1.43 | 1.54 | 2.22 | 15.03 | 1.29 | 1.76 |
| Firmicutes | Gemella haemolysans | 0.00 | 0.00 | 0.00 | 0.00 | 0.00 | 0.14 | 0.04 | 0.60 | 0.00 | 0.14 | 0.00 | 0.28 | 0.00 | 0.00 | 0.00 | 0.00 | 0.05 | 0.35 |
| Firmicutes | Gemella sanguinis | 0.00 | 0.00 | 0.17 | 0.00 | 0.00 | 0.00 | 0.00 | 0.00 | 0.00 | 0.00 | 0.22 | 0.40 | 0.22 | 0.00 | 0.00 | 0.00 | 0.17 | 0.21 |
| Firmicutes | Lachnoanaerobaculum saburreum | 0.00 | 0.10 | 0.00 | 0.20 | 0.12 | 0.16 | 0.00 | 0.30 | 0.00 | 0.14 | 0.00 | 0.00 | 0.00 | 1.29 | 0.00 | 0.00 | 0.14 | 0.34 |
| Firmicutes | Megasphaera micronuciformis | 1.08 | 0.00 | 1.13 | 0.00 | 0.44 | 0.00 | 0.53 | 0.33 | 0.28 | 0.05 | 1.34 | 0.00 | 0.14 | 0.00 | 0.00 | 0.00 | 0.61 | 0.30 |
| Firmicutes | Mitsuokella sp. oral taxon 131 | 0.00 | 0.00 | 0.00 | 0.00 | 0.00 | 0.00 | 0.28 | 0.00 | 0.00 | 0.00 | 0.65 | 0.00 | 0.00 | 0.09 | 0.00 | 0.00 | 0.40 | 0.07 |
| Firmicutes | Oribacterium parvum | 0.00 | 0.20 | 0.17 | 0.25 | 0.50 | 0.16 | 0.51 | 0.00 | 0.26 | 0.00 | 0.00 | 0.20 | 0.73 | 0.00 | 0.00 | 0.00 | 0.42 | 0.13 |
| Firmicutes | Oribacterium sinus | 0.40 | 0.98 | 1.37 | 0.22 | 1.47 | 0.00 | 0.11 | 0.40 | 0.00 | 0.73 | 3.30 | 3.09 | 3.76 | 0.31 | 18.05 | 0.76 | 2.42 | 0.61 |
| Firmicutes | Peptostreptococcus stomatis | 0.16 | 0.22 | 1.03 | 0.61 | 1.17 | 0.29 | 1.25 | 0.53 | 0.99 | 0.50 | 0.30 | 0.99 | 0.69 | 0.14 | 0.00 | 0.88 | 0.79 | 0.38 |
| Firmicutes | Selenomonas flueggei | 23.50 | 2.36 | 6.31 | 0.64 | 5.45 | 2.08 | 3.64 | 0.92 | 9.30 | 0.67 | 11.47 | 0.00 | 2.29 | 1.27 | 4.57 | 0.00 | 6.61 | 1.12 |
| Firmicutes | Selenomonas infelix | 1.34 | 1.60 | 1.18 | 0.23 | 0.50 | 0.16 | 0.37 | 0.00 | 0.00 | 0.00 | 0.29 | 0.00 | 2.66 | 0.00 | 0.98 | 0.00 | 0.82 | 0.38 |
| Firmicutes | Selenomonas noxia | 0.00 | 0.15 | 0.24 | 0.00 | 0.40 | 0.00 | 0.33 | 0.00 | 0.68 | 0.00 | 0.00 | 0.00 | 0.00 | 0.13 | 0.00 | 0.00 | 0.43 | 0.10 |
| Firmicutes | Selenomonas sp. oral taxon 126 | 1.41 | 0.00 | 0.31 | 0.00 | 0.26 | 0.00 | 0.27 | 0.04 | 0.85 | 0.00 | 0.23 | 0.11 | 0.41 | 0.00 | 0.00 | 0.00 | 0.45 | 0.06 |
| Firmicutes | Selenomonas sp. oral taxon 149 | 0.00 | 0.00 | 1.02 | 0.00 | 0.00 | 0.00 | 0.00 | 0.00 | 0.00 | 0.00 | 1.52 | 0.00 | 2.09 | 0.00 | 6.13 | 0.00 | 1.65 | 0.00 |
| Firmicutes | Selenomonas sp. oral taxon 478 | 0.00 | 0.00 | 0.74 | 0.00 | 0.34 | 0.00 | 0.32 | 0.00 | 0.27 | 0.00 | 0.33 | 0.00 | 0.99 | 0.00 | 0.00 | 0.00 | 0.46 | 0.00 |
| Firmicutes | Selenomonas sp. oral taxon 920 | 0.85 | 0.00 | 1.03 | 0.00 | 1.20 | 0.31 | 1.24 | 0.35 | 1.88 | 0.10 | 0.62 | 0.96 | 0.40 | 0.18 | 0.00 | 0.00 | 0.99 | 0.32 |
| Firmicutes | Selenomonas sputigena | 1.42 | 0.00 | 1.69 | 0.00 | 2.90 | 0.18 | 0.69 | 0.05 | 1.47 | 0.00 | 3.09 | 0.00 | 1.70 | 0.00 | 7.62 | 0.00 | 1.90 | 0.11 |
| Firmicutes | Solobacterium moorei | 0.00 | 0.00 | 0.50 | 0.00 | 0.40 | 0.00 | 0.05 | 0.00 | 0.68 | 0.00 | 0.12 | 0.00 | 0.12 | 0.00 | 0.00 | 0.00 | 0.31 | 0.00 |
| Firmicutes | Stomatobaculum longum | 0.00 | 0.00 | 0.33 | 0.36 | 2.14 | 0.00 | 0.35 | 0.41 | 0.00 | 0.00 | 1.21 | 0.36 | 0.44 | 0.00 | 0.98 | 1.76 | 0.81 | 0.39 |
| Firmicutes | Streptococcus mitis | 0.00 | 1.97 | 1.25 | 2.77 | 0.51 | 3.72 | 0.00 | 1.98 | 0.23 | 2.10 | 0.00 | 8.73 | 0.09 | 2.38 | 0.00 | 11.30 | 0.52 | 2.64 |
| Firmicutes | Veillonella atypica | 2.16 | 0.00 | 0.50 | 0.00 | 0.32 | 0.00 | 0.16 | 0.00 | 0.69 | 0.00 | 3.25 | 0.00 | 0.07 | 0.00 | 0.00 | 0.00 | 0.81 | 0.00 |
| Firmicutes | Veillonella dispar | 0.00 | 0.26 | 0.00 | 0.00 | 0.07 | 0.00 | 0.21 | 0.00 | 0.06 | 0.00 | 0.00 | 0.00 | 0.00 | 0.00 | 0.00 | 0.00 | 0.12 | 0.14 |
| Firmicutes | Veillonella sp. oral taxon 158 | 0.00 | 0.00 | 0.04 | 0.23 | 0.00 | 0.19 | 0.00 | 0.00 | 0.00 | 0.22 | 0.31 | 0.00 | 0.00 | 0.18 | 0.00 | 0.63 | 0.14 | 0.18 |
| Fusobacteria | Fusobacterium nucleatum | 0.00 | 17.94 | 0.00 | 0.00 | 0.05 | 17.10 | 0.00 | 8.05 | 0.05 | 7.31 | 0.04 | 0.00 | 0.03 | 20.49 | 0.00 | 0.00 | 0.04 | 12.48 |
| Fusobacteria | Fusobacterium periodonticum | 0.00 | 0.70 | 0.20 | 1.11 | 0.13 | 0.53 | 0.18 | 0.69 | 0.15 | 0.80 | 0.19 | 1.30 | 0.12 | 0.78 | 0.00 | 0.00 | 0.15 | 0.70 |
| Fusobacteria | Fusobacterium sp. oral taxon 370 | 0.00 | 0.00 | 0.23 | 0.00 | 0.34 | 0.00 | 0.46 | 0.00 | 0.51 | 0.00 | 0.27 | 0.00 | 0.22 | 0.00 | 0.00 | 0.00 | 0.33 | 0.00 |
| Fusobacteria | Leptotrichia buccalis | 0.00 | 0.43 | 0.00 | 2.46 | 0.00 | 1.86 | 0.00 | 0.17 | 0.00 | 0.46 | 0.00 | 1.88 | 0.00 | 2.00 | 0.00 | 0.00 | 0.00 | 0.96 |
| Fusobacteria | Leptotrichia sp. oral taxon 212 | 0.00 | 0.00 | 0.00 | 0.70 | 0.00 | 0.52 | 0.00 | 0.31 | 0.13 | 0.00 | 0.11 | 0.91 | 0.21 | 0.50 | 0.00 | 0.00 | 0.13 | 0.45 |
| Fusobacteria | Leptotrichia sp. oral taxon 215 | 0.39 | 1.20 | 0.30 | 1.85 | 0.62 | 1.31 | 0.83 | 0.37 | 0.60 | 0.88 | 1.29 | 0.86 | 0.18 | 0.58 | 0.00 | 0.00 | 0.55 | 0.78 |
| Fusobacteria | Leptotrichia sp. oral taxon 847 | 0.00 | 0.00 | 0.00 | 0.00 | 0.00 | 0.00 | 0.00 | 0.00 | 0.00 | 0.00 | 0.00 | 0.00 | 0.00 | 0.00 | 0.00 | 0.00 | 0.00 | 0.00 |
| Fusobacteria | Leptotrichia wadei | 0.00 | 0.18 | 0.10 | 0.18 | 0.07 | 0.17 | 0.00 | 0.17 | 0.08 | 0.19 | 0.00 | 0.99 | 0.13 | 0.14 | 0.00 | 0.67 | 0.09 | 0.21 |
| Proteobacteria | Aggregatibacter segnis | 0.00 | 0.16 | 0.00 | 0.24 | 0.27 | 0.36 | 0.27 | 0.00 | 0.17 | 0.14 | 0.00 | 0.00 | 0.00 | 0.00 | 0.00 | 0.00 | 0.25 | 0.17 |
| Proteobacteria | Aggregatibacter sp. oral taxon 458 | 0.00 | 2.07 | 0.00 | 3.54 | 0.14 | 1.06 | 0.32 | 0.00 | 0.00 | 0.88 | 0.00 | 3.21 | 0.00 | 1.64 | 0.00 | 1.74 | 0.24 | 1.24 |
| Proteobacteria | Campylobacter concisus | 0.66 | 0.00 | 0.81 | 0.00 | 0.89 | 0.00 | 0.49 | 0.00 | 0.67 | 0.15 | 4.16 | 0.15 | 1.46 | 0.00 | 0.00 | 0.00 | 1.13 | 0.13 |
| Proteobacteria | Campylobacter rectus | 0.28 | 0.00 | 0.16 | 0.00 | 0.16 | 0.15 | 0.37 | 0.04 | 0.00 | 0.00 | 0.07 | 0.00 | 0.57 | 0.00 | 0.00 | 0.00 | 0.23 | 0.09 |
| Proteobacteria | Campylobacter showae | 0.77 | 0.77 | 1.68 | 1.03 | 1.69 | 0.79 | 0.21 | 0.64 | 0.44 | 0.68 | 1.85 | 0.98 | 0.78 | 0.45 | 0.39 | 1.19 | 0.87 | 0.59 |
| Proteobacteria | Cardiobacterium hominis | 0.00 | 1.06 | 0.36 | 1.36 | 0.10 | 1.21 | 0.38 | 0.83 | 0.48 | 0.90 | 0.13 | 0.28 | 0.29 | 1.18 | 0.00 | 0.00 | 0.28 | 0.85 |
| Proteobacteria | Cardiobacterium valvarum | 0.00 | 0.16 | 0.00 | 0.27 | 0.00 | 0.38 | 0.08 | 0.08 | 0.00 | 0.53 | 0.00 | 0.37 | 0.00 | 0.36 | 0.00 | 1.03 | 0.08 | 0.26 |
| Proteobacteria | Delftia acidovorans | 0.00 | 2.35 | 1.10 | 0.29 | 2.08 | 0.00 | 1.99 | 0.58 | 3.63 | 0.29 | 0.00 | 0.92 | 0.00 | 0.39 | 0.00 | 8.66 | 2.30 | 0.84 |
| Proteobacteria | Desulfomicrobium orale | 0.82 | 0.00 | 0.28 | 2.55 | 0.38 | 0.00 | 0.60 | 0.00 | 0.45 | 0.00 | 0.16 | 0.00 | 0.06 | 0.00 | 0.00 | 0.00 | 0.35 | 1.56 |
| Proteobacteria | Haemophilus parainfluenzae | 0.00 | 0.00 | 0.13 | 0.21 | 0.15 | 0.00 | 0.17 | 0.07 | 0.17 | 0.13 | 0.00 | 0.00 | 0.00 | 0.32 | 0.00 | 0.00 | 0.16 | 0.17 |
| Proteobacteria | Haemophilus sp. CCUG 60358 | 0.00 | 0.00 | 0.11 | 0.00 | 0.00 | 0.00 | 0.00 | 0.39 | 0.30 | 0.13 | 0.00 | 0.85 | 0.00 | 0.00 | 0.00 | 1.56 | 0.21 | 0.42 |
| Proteobacteria | Kingella denitrificans | 0.00 | 2.84 | 0.30 | 3.74 | 0.00 | 2.22 | 0.00 | 49.10 | 0.00 | 44.52 | 0.00 | 0.00 | 0.00 | 1.71 | 0.00 | 0.00 | 0.28 | 23.45 |
| Proteobacteria | Lautropia mirabilis | 4.37 | 4.58 | 9.12 | 7.81 | 5.96 | 5.90 | 17.04 | 3.23 | 16.31 | 2.54 | 10.02 | 7.49 | 7.94 | 3.33 | 0.43 | 10.01 | 8.39 | 3.72 |
| Proteobacteria | Neisseria bacilliformis | 0.45 | 0.00 | 0.00 | 0.00 | 0.00 | 0.52 | 0.00 | 0.41 | 0.21 | 0.28 | 0.54 | 0.00 | 0.00 | 0.00 | 0.00 | 0.00 | 0.31 | 0.47 |
| Proteobacteria | Neisseria cinerea | 0.00 | 0.00 | 0.07 | 0.30 | 0.05 | 0.16 | 0.00 | 0.08 | 0.00 | 0.00 | 0.00 | 0.00 | 0.00 | 0.21 | 0.00 | 0.00 | 0.06 | 0.16 |
| Proteobacteria | Neisseria elongata | 0.55 | 2.84 | 0.29 | 1.26 | 0.83 | 2.39 | 2.08 | 0.35 | 0.86 | 0.42 | 0.10 | 0.18 | 0.00 | 1.65 | 0.00 | 1.13 | 0.78 | 0.88 |
| Proteobacteria | Neisseria flavescens | 4.85 | 4.73 | 1.15 | 8.17 | 4.35 | 8.01 | 6.71 | 5.31 | 2.29 | 4.76 | 2.08 | 2.84 | 0.42 | 14.60 | 0.00 | 0.69 | 2.89 | 5.27 |
| Proteobacteria | Neisseria mucosa | 0.00 | 3.57 | 2.77 | 0.22 | 7.96 | 3.99 | 0.44 | 3.14 | 7.18 | 7.69 | 0.00 | 11.01 | 2.91 | 0.91 | 0.00 | 6.98 | 4.41 | 3.51 |
| Proteobacteria | Neisseria sicca | 0.00 | 0.00 | 0.00 | 0.25 | 0.00 | 0.22 | 0.00 | 0.09 | 0.00 | 0.00 | 0.00 | 0.70 | 0.00 | 0.21 | 0.00 | 0.00 | 0.00 | 0.20 |
| Proteobacteria | Neisseria sp. oral taxon 014 | 8.62 | 7.25 | 4.24 | 12.64 | 3.17 | 7.85 | 10.18 | 0.73 | 4.47 | 0.60 | 3.41 | 0.13 | 0.33 | 8.34 | 6.84 | 0.96 | 4.23 | 3.39 |
| Proteobacteria | Ottowia sp. oral taxon 894 | 0.00 | 1.41 | 0.15 | 1.39 | 0.10 | 0.82 | 0.52 | 0.78 | 0.18 | 0.36 | 0.00 | 0.00 | 0.00 | 1.30 | 0.00 | 0.73 | 0.24 | 0.75 |
| Proteobacteria | Ralstonia pickettii | 1.92 | 0.73 | 0.57 | 0.46 | 1.24 | 0.51 | 4.47 | 0.00 | 1.87 | 0.00 | 0.37 | 0.00 | 0.00 | 0.72 | 0.00 | 0.38 | 1.65 | 0.36 |
| Spirochaetes | Treponema vincentii | 0.00 | 0.00 | 0.25 | 0.00 | 0.60 | 0.00 | 0.00 | 0.00 | 1.14 | 0.05 | 1.17 | 0.00 | 0.11 | 0.00 | 1.10 | 0.00 | 0.60 | 0.06 |
| Synergistetes | Fretibacterium fastidiosum | 7.23 | 2.21 | 0.61 | 8.74 | 2.19 | 7.16 | 5.53 | 0.00 | 3.54 | 0.00 | 4.44 | 0.00 | 0.25 | 2.23 | 0.00 | 0.00 | 2.94 | 3.55 |

Table S5. Primer sequences for qPCR verification

| **Taxonomy** | **Primer sequences** |
| --- | --- |
|  |  |
| *Actinomyces graevenitzii* | F: CTGAGATACGGCCCAGACTC |
|  | R: CACCTTCCTCCGAGTTGACC |
| *Capnocytophaga* sp. oral taxon 329 | F: ATACCCTGGTAGTCCACGCT |
|  | R: CTTGGTAAGGTTCCTCGCGT |
| *Fusobacterium nucleatum* | F: GCTAAATACGTGCCAGCAGC |
|  | R: CTGGTAAGGTTCCTCGCGTT |
| *Kingella denitrificans* | F: TGACGTCAAGTCCTCATGGC |
|  | R: TTTCTGGGATTGGCTCCACC |
| *Prevotella histicola* | F: TCACTGACGGCATCAGATGTG |
|  | R: GGCTGGTTCAGGCTCTCGC |
| *Selenomonas* sp. oral taxon 126 | F: AGCTGGTTGGTGAGGTAACG |
|  | R: AAAGGGTTATTCCCCTTCGCA |
| *Streptococcus mitis* | F: GAACGCTGAAGGAGGAGCTT |
|  | R: GTGTCTCAGTCCCAGTGTGG |
| Universal primer | F: GTGSTGCAYGGYTGTCGTCA |
|  | R: ACGTCRTCCMCACCTTCCTC |
